# Design, synthesis, and biological investigations of new pyrazole derivatives as VEGFR2/CDK-2 inhibitors targeting liver cancer

**DOI:** 10.1186/s13065-024-01314-z

**Published:** 2024-10-24

**Authors:** Manar G. Salem, Mohamed S. Nafie, Aya A. Elzamek, Hosam A. Elshihawy, Mamdouh A. Sofan, Elham Negm

**Affiliations:** 1 Pharmaceutical Organic Chemistry Department, Faculty of Pharmacy, Suez Canal University, Ismailia, P.O. 41522 Egypt; 2 Department of Chemistry, College of Sciences, University of Sharjah, P.O. 27272 Sharjah, United Arab Emirates; 3 Chemistry Department, Faculty of Science, Suez Canal University, Ismailia, P.O 41522 Egypt; 4 Department of Chemistry, Faculty of Science, Damietta University, New Damietta, Egypt

**Keywords:** Pyrazole, Anti-cancer activity, VEGFR2, CDK-2 kinase activities, Molecular docking

## Abstract

New Series of *N*-Manniche bases **3,4 (a-c)** and **5,6 (a-b)** were synthesized through the reaction of benzaldehyde and amine with 3-methyl-4-(aryldiazenyl)-1H-pyrazol-5-ol derivatives **2(a-c),** they were fully characterized by FT-IR, (^1^H, ^13^C) NMR data in addition to their mass spectra. The Structural Activity Relationship of the target compounds were examined for their cytotoxicity. Some newly synthesized compounds showed promising antiproliferation properties when tested against HepG2 cancer cells. Compounds **4a, 5a,** and **6b** showed potent cytotoxicity against HepG2 with IC_50_ values of 4.4, 3.46 and 2.52 µM compared to Sorafenib (IC_50_ = 2.051 µM) and Roscovitine (IC_50_ = 4.18 µM). Furthermore, they were safe against the THLE2 cells with higher IC_50_ values. Compound **6b** exhibited promising dual VEGFR2/CDK-2 inhibition activities; it had an IC_50_ value of 0.2 μM with VEGFR2 inhibition of 93.2%, and it had an IC_50_ value of 0.458 μM with CDK-2 inhibition of 88.7%. In comparison to the untreated control group (0.95%), compounds **5a** (38.32%) and **6b** (42.9%) considerably increased the cell population in total apoptosis. In addition, compounds **5a** and **6b** arrested the cell population at G0-G1 and S phases, respectively. Molecular docking experiments confirmed the virtual binding mechanism of the most active drugs, which were found to have good binding affinities with both receptor active sites.

**Supplementary Information:**

The online version contains supplementary material available at 10.1186/s13065-024-01314-z.

## Introduction

**Fig. 1:**
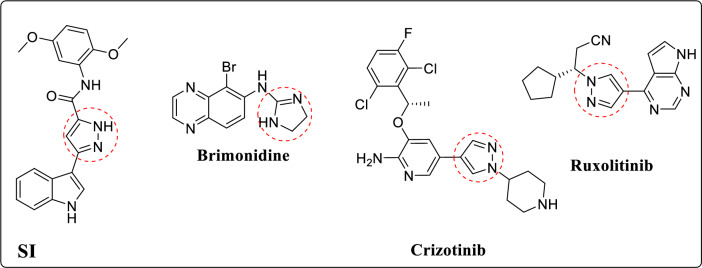
Pyrazole-based anti-cancer agents

Cancer remains a significant global health issue owing to its close relation with approximately 100 diseases, affecting many organs in the body [1]. To date, it is the second leading cause of mortality globally [1–4]. The primary route begins when a cell multiplies and grows abnormally beyond what is considered normal [1, 5, 6]. Liver cancer is considered one of the extremely common malignant tumors in the gastrointestinal system. Currently, Sorafenib is the only drug that has FDA approval for the treatment of hepatocellular carcinoma (HCC), which can only extend patient survival for a few months [7]. However, the typical clinical treatments available, like surgery, radiotherapy, and chemotherapy, regularly end in adverse side effects. Moreover, liver cancer cells display inherent resistance to standard chemotherapy and radiotherapy [8]. Hence, innovative schemes that provide both better efficacy and reduced side effects are of great demand [9–11]. Recently, there have been significant advances in the understanding and targeting of numerous pathways crucial to the development of cancer therapies [12]. It is common knowledge that DNA function is disrupted by conventional anti-cancer medicines. Some of these medicines may interfere with DNA synthesis by blocking crucial enzymes [13–15]. Cyclin-dependent kinase 2 (CDK-2) is a serine/threonine kinase that regulates the transition from the G1 to the S phase of the cell cycle [16]. Studies have shown that CDK-2 promotes apoptosis in addition to its role in cell cycle progression, albeit the underlying mechanism of this paradoxical function is yet unknown. Accordingly, inhibiting the CDK-2 enzyme could result in G1/S and G2/M cell cycle phase arrest and apoptosis induction [17]. The most important angiogenic factor is vascular endothelial growth factor receptor 2, or VEGFR2; it is a type III receptor tyrosine kinase that binds to tumor-secreted vascular endothelial growth factor (VEGF) and becomes highly activated on vascular endothelial cells upon interaction [18]. Cancers of the liver, stomach, colon, lungs, and breasts are among the many solid-type human malignancies that show overexpression of VEGFR2, a protein crucial for cell death [19]. Active VEGFR2 facilitates the construction of a blood vessel network for the tumor cells [20]. Consequently, VEGFR2 inhibition has become a promising strategy for the treatment of many types of cancer, including liver cancers [21]. Pyrazole-based derivatives possess various anti-cancer bioactivities [22, 23]. Ruxolitinib, Brimonidine, and Crizotinib are three examples of anti-cancer drugs available in the market and based on pyrazole moiety. Besides, compound **SI** showed potent activity against the HepG2 liver cancer cells, with IC_50_ value 0.6 μM (Fig. 1) [18, 24, 25].

**Fig. 2:**
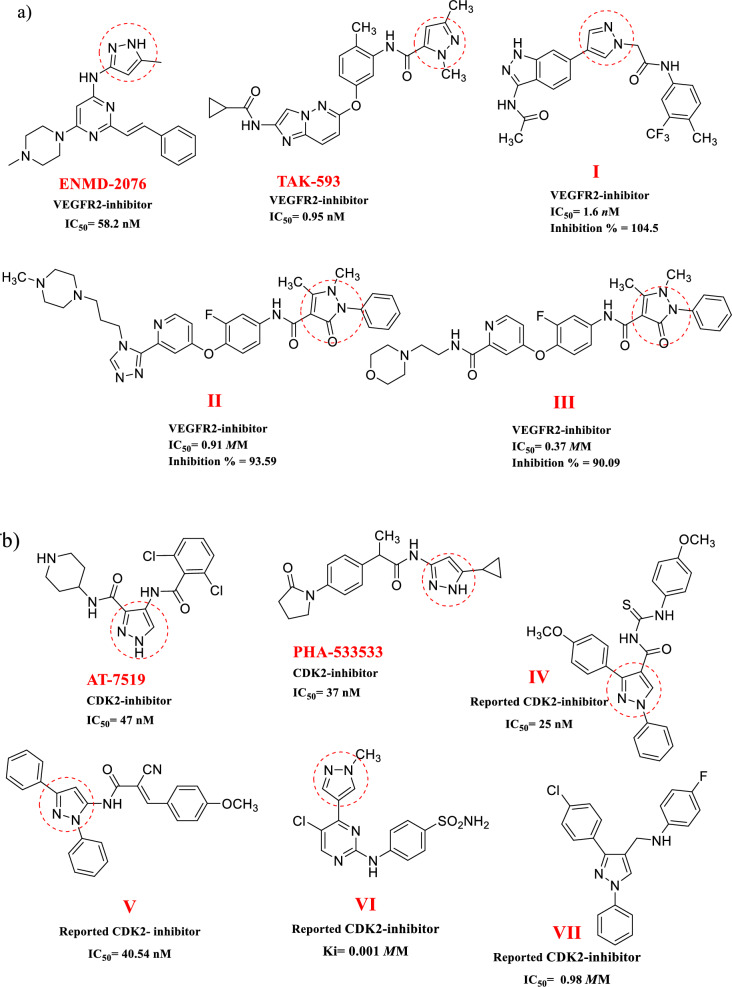
Pyrazole-based anti-cancer compounds; **a** pyrazole derivatives as VEGFR2 inhibitors [18, 26, 28]. **b** pyrazole derivatives as CDK-2 inhibitors[7, 27, 29]

A common pharmacophoric factor for inhibiting the activity of the VEGFR2 and CDK-2 enzymes, according to the reviewed literature, is the pyrazole template (Fig. 2a, b) [21, 26, 27].

**Fig. 3:**
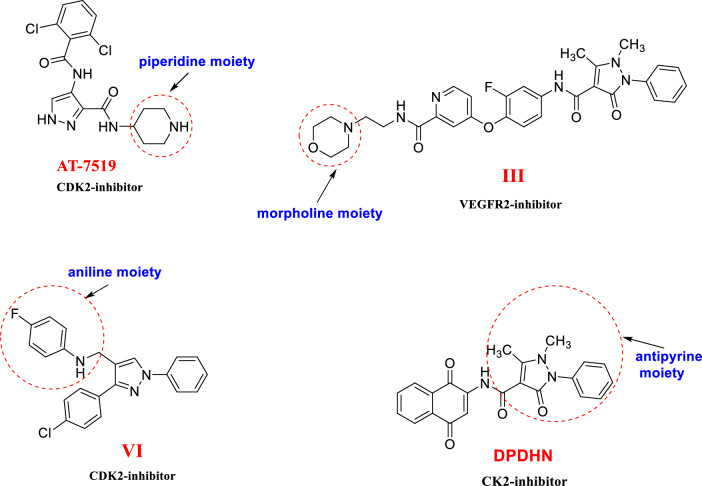
Anti-cancer agents with reactive species: piperidine, morpholine, aniline, and antipyrine [27, 31, 32]

Although pyrazole analogs have proven great potency as anti-cancer agents, a few of them have been withdrawn from the market owing to their side effects (such as bone marrow depression) and drug resistance. Therefore, research on potent new drug applicants bearing pyrazole scaffold with high specificity and lesser side effects has increased recently. The pyrazole nucleus is the building block of the pyrazolone molecule, which had an additional carbonyl (C=O) group. Compounds containing this functional group are beneficial commercially and are considered the basis of several pharmaceuticals [21]. The discovery of pyrazolone efficacy led us to synthesize new pyrazolone derivatives with similar properties but improved therapeutic action through the introduction of another active heterocyclic moiety with high potencies like piperidine, morpholine, aniline, and antipyrine based on a hybridization strategy and a mixed pharmacophore theory [19, 30]. Figure 3 illustrates some reported anti-tumor compounds bearing piperidine, morpholine, aniline, or antipyrine as reactive moieties.

**Fig. 4:**
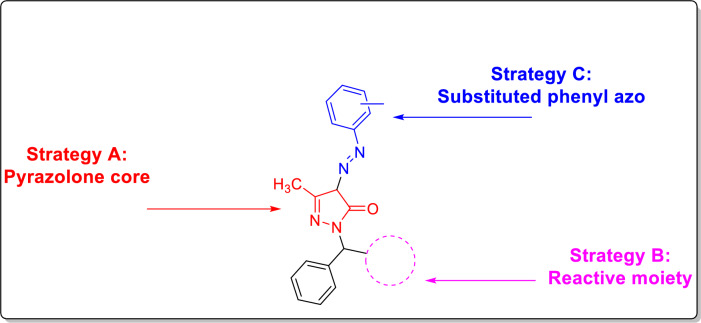
The proposed scaffolds of VEGFR2 & CDK-2 inhibitors

We aimed to synthesize novel *N*-mannich pyrazole-5-ol derivatives and test their ability to inhibit VEGFR2 and CDK-2 kinases based on a mixed pharmacophore theory (Fig. 4).

The anti-cancer activity was evaluated simultaneously using cancer cells expressing enzymes. In addition, a molecular modeling study was conducted to investigate the optimal binding modes of the most effective target compounds that matched the binding modes of the ligands.

## Materials and methods

### Chemistry

Sigma Aldrich company was the main source for all the chemicals and used as received. The determination of all melting points was achieved through open capillary tubes with a Griffin melting point apparatus and was uncorrected. FTIR (cm^−1^) spectra were recorded on KBr pellets using a JASCO 410 spectrometer, with only selected absorptions recorded in the range of (4000–400) cm^−1^. (^1^H-^13^C) NMR spectra were measured in deuterated dimethyl sulfoxide (DMSO-d_6_) using a Bruker Advance III 400 MHz NMR spectrometer and a JEOL ECA-II 500 MHz NMR spectrometer, respectively. The coupling constants (*J*) are given in hertz. As an internal reference, the expression of chemical shifts is stated as δ values in ppm downfield from tetramethyl silane. Mass spectra were recorded on Kratos (70eV) MS equipment and/or a Varian MAT 311A Spectrometer. Elemental analyses were achieved using PerkinElmer 240 “Cairo University, Cairo, Egypt”. All reactions were followed by TLC (performed using 0.2 mm precoated plates of silica gel G60 F, Merck). TLC was visualized by UV light (254 and 366 nm) or with iodine vapor.

#### A general method for preparation of compounds 2(a-c)

A sodium nitrite solution (12 mmol) was used to diazotize an arylamine (10 mmol) solution in Conc. HCl (5 ml, 6 M) at 0–5 °C. The resulting diazonium salt solution was stirred into a solution of ethyl acetoacetate (10 mmol) in 20 ml of ethanol containing sodium acetate (3 g, 35 mmol) after keeping it at 0–5°c for an hour. After an hour of stirring the mixture, the crude product **1(a-c)** was filtered and purified with ethanol [33–35]. After that, for four hours, a mixture of hydrazine hydrate (20 mmol) and 3-(aryldiazenyl)pentane-2,4-dione derivatives **1(a-c)** (10 mmol) in 15 ml of absolute ethanol were refluxed. The reaction mixture was chilled and dispensed into ice-cold water with stirring. The precipitated compound was filtered and recrystallized from ethanol to afford **2(a-c) **[36]**.**

*5-Methyl-4-(phenyldiazenyl)-2,4-dihydro-3H-pyrazol-3-one (2a)* Yellow crystals; 85% yield; m.p (186–188)°C; was characterized by thin layer chromatography (TLC) Rf = 0.68 eluent system (ethyl acetate: petroleum ether 1:3 v/v on silica gel); IR (ν/cm^−1^): 1445.36 -1482.03 (N = N), 1548.55 (C = C_Aromatic_), 1661.37 (C = O), 3064.33 (CH), 3169.44 (NH), 3304.4(OH); ^1^H-NMR (500 MHz, DMSO- d_6_): δ 2.15 (3H, S, CH_3_), 7.16 (1H, m, Ar–H), 7.40 (2H, m, Ar–H), 7.51 (2H, m, Ar–H), 11.56 (1H, s, OH_exchange with D2O_), 12.88 (1H, s, NH_exchange with D2O_).

*5-Methyl-4-((2-nitrophenyl)diazenyl)-2,4-dihydro-3H-pyrazol-3-one (2b)* Yellow crystals; 80% yield; m.p (250–252)°C; was characterized by thin layer chromatography (TLC) Rf = 0.69 eluent system (E.A: P.E = 1:2 v/v on silica gel); IR (ν/cm^−1^): 1438.64–1492.63 (N = N), 1575.59 (C = C_Aromatic_), 1679.69 (C = O), 3200.2 (NH), 3408.5 (OH); ^1^H-NMR (500 MHz, DMSO): δ 2.89 (3H, S, CH_3_), 6.86–8.27 (4H, m, Ar–H), 11.81 (1H, s, OH_exchange with D2O_), 14.40 (1H, s, NH_exchange with D2O_).

*4-((4-Chlorophenyl)diazenyl)-5-methyl-2,4-dihydro-3H-pyrazol-3-one(2c)* Yellow crystals; 89% yield; m.p (228–230)°C; was characterized by thin layer chromatography (TLC) Rf = 0.75 eluent system (E.A: P.E = 1:5 v/v on silica gel); IR (ν/cm^−1^): 1380.78–1479.13 (N = N), 1559.17 (C = C_Aromatic_), 1667.16 (C = O), 3218.6 (NH), 3445.2 (OH); ^1^H-NMR (500 MHz, DMSO-d_6_): δ 2.13(3H, s, CH_3_), 7.45 (2H, d, ^*3*^*J* = 7.7 Hz, Ar–H), 7.56 (2H, d, ^*3*^*J* = 7.8 Hz, Ar–H), 11.59 (1H, s, OH_exchange with D2O_), 13.12 (1H, s, NH_exchange with D2O_).

#### A general method for preparation of compounds 3,4 (a-c)

In a steam bath, a mixture of compound **2(a-c)** (10 mmol), morpholine or piperidine (15 mmol), benzaldehyde (10 mmol), and ethanol (20 ml) was heated for 2 h. and then stirred for 24 h at room temperature. The precipitated compound was filtered and recrystallized from ethanol to obtain **3,4 (a-c) **[37].

*5-Methyl-2-(morpholino(phenyl)methyl)-4-(phenyldiazenyl)-2,4-dihydro-3H-pyrazol-3-one: (3a)* Yellow crystals; 88% yield; m.p (156–158)°C; IR (ν/cm^−1^): 1560.1 (C = C_Aromatic_), 3434.6 (OH); ^1^H-NMR (500 MHz, DMSO-d_6_): δ 2.2 (3H, s, CH_3_), 2.51- 2.54 (4H, m, CH_2_^*^-N), 3.55- 3.61 (4H, m, CH_2_^*^-O), 5.90 (1H, s, Ar–CH*-N), 7.19 7.56 ( 10H, m, Ar–H), 12.96 (1H, s, OH_exchange with D2O_). ^13^C-NMR (125.6 MHz, DMSO-d_6_): 11.83, 49.33, 66.2, 87.86, 116.19, 125.61, 128.24, 128.43, 128.86, 129.13, 130.12, 135.14, 137.06, 158.98, 160.70. EI-MS (m/z, %): 377.61 [M^+^]. Calc. for **C**_**21**_**H**_**23**_**N**_**5**_**O**_**2**_: C, 66.83; H, 6.14; N, 18.55. Found: C, 66.69; H, 6.31; N, 18.83.

*5-Methyl-2-(morpholino(phenyl)methyl)-4-((2-nitrophenyl)diazenyl)-2,4-dihydro-3H-pyrazol-3-one: (3b)* orange crystals; 75% yield; m.p (182–184)°C; IR (ν/cm^−1^): 3438.46 (OH); ^1^H-NMR (500 MHz, DMSO-d_6_): δ 2.25 (3H, s, CH_3_*), 2.77- 2.79 (2H, m), 3.53- 3.55 (6H, m), 5.95 (1H, s, Ar–CH*-N), 7.35–7.38 (3H, m, Ar–H),7.43- 7.45 (1H, t, ^*3*^*J* = 7.6 Hz, Ar–H), 7.69–7.72 (1H, t, ^*3*^*J* = 7.8 Hz, Ar–H), 7.89–7.91 (3H, m, Ar–H), 8.11–8.15 (1H, d, ^*3*^* J* = 8.1 Hz, Ar–H), 11.79 (1H, s, OH_exchange with D2O_). ^13^C-NMR (125.6 MHz, DMSO-d_6_): 12.34, 49.83, 66.77, 74.37, 116.97, 117.15, 124.76, 126.50, 128.47, 128.89, 129.70, 130.03, 135.14, 135.51, 136.75, 137.03, 147.39, 158.45. EI-MS (m/z, %): 422.14 [M^+^]. Calc. for **C**_**21**_**H**_**22**_**N**_**6**_**O**_**4**:_ C, 59.71; H, 5.25; N, 19.89. Found: C, 59.98; H, 5.41; N, 20.07.

*4-((4-Chlorophenyl)diazenyl)-5-methyl-2-(morpholino(phenyl)methyl)-2,4-dihydro-3H-pyrazol-3-one (3c)* Yellow crystals; 79% yield; m.p (192–194)°C; IR (ν/cm^−1^): 1450.2–1483.9 (N=N), 1563.9 (C=C), 1657.5 (C=O), 3437.4 (OH); ^1^H-NMR (500 MHz, DMSO-d_6_): δ 2.20 (3H, s, CH_3_), 2.55–257 (4H, m), 3.54–3.59 (4H, m), 5.90 (1H, s, Ar–CH*-N), 7.36–7.50 (4H, m, Ar–H),7.56–60 (2H, m, Ar–H), 7.61- 7.63 (1H, m, Ar–H), 7.71–7.73 (2H, d, ^*3*^* J* = 8 Hz, Ar–H), 11.58 (1H, s, OH_exchange with D2O_). ^13^C-NMR (125.6 MHz, DMSO-d_6_): 12.20, 45.76, 49.28, 49.68, 88.36, 117.94, 118.38, 128.22, 128.42, 129.13, 129.71, 129.94, 134.29, 135.15, 193.83. EI-MS (m/z, %): 411.72 [M^+^], 413.81 [M^+2^]. Calc. for **C**_**21**_**H**_**22**_**ClN**_**5**_**O**_**2**_: C, 61.24; H, 5.38; N, 17.00. Found: C, 61.38; H, 5.60; N, 17.23.

*5-Methyl-2-(phenyl(piperidin-1-yl)methyl)-4-(phenyldiazenyl)-2,4-dihydro-3H -pyrazol-3-one (4a)* Yellow crystals; 65% yield; m.p (132–134)°C; IR (ν/cm^−1^): 3424.69 (OH); ^1^H-NMR (500 MHz, DMSO—d_6_): δ 1.30–1.58 (10H, m), 2.20 (2H, s, CH_3_), 5.96 (1H, s, Ar–CH*-N), 7.16–7.62 (8H, m, Ar–H), 7.72–7.92 (2H, dd, *J* = 8.3, 1.5 Hz, Ar–H),12.47 (1H, s, OH_exchange with D2O_). ^13^C-NMR (125.6 MHz, DMSO-d_6_): 12.43, 23.03, 23.82, 26.23, 50.54, 116.29, 116.87, 125.56, 127.97, 128.15, 128.68, 129.70, 130.4, 135.14, 136.69, 193.81. EI-MS (m/z, %): 374.92 [M^+^]. Calc. for **C**_**22**_**H**_**25**_**N**_**5**_**O**: C, 70.38; H, 6.71; N, 18.65. Found: C, 70.15; H, 6.82; N, 18.89.

*5-Methyl-4-((2-nitrophenyl)diazenyl)-2-(phenyl(piperidin-1-yl)methyl)-2,4-dihydro-3H-pyrazol-3-one (4b)* orange crystals; 71% yield; m.p (162–164)°C; IR (ν/cm^−1^): 3438.4 (OH); ^1^H-NMR (500 MHz, DMSO- d_6_): δ 1.35–1.57 (6H, m), 2.21 (3H, s), 2.51–2.54 (4H, m), 5.99 (1H, s, Ar–CH-N), 7.32–7.38 (5H, m, Ar–H), 7.57–7.59 (1H, m, Ar–H), 7.61–7.86 (1H, m, Ar–H), 8.11–8.14 (1H, m, Ar–H), 8.21–8.26 (1H, m, Ar–H), 11.78 (1H, s, OH_exchange with D2O_). ^13^C-NMR (125.6 MHz, DMSO-d_6_): 12.24, 23.19, 24.14, 40.85, 45.24, 40.85, 45.24, 116.29, 116.87, 125.56, 127.97, 128.15, 128.68, 129.70, 130.04, 135.14, 136.69, 193.86. EI-MS (m/z, %): 421.19 [M^+^]. Calc. for **C**_**22**_**H**_**24**_**N**_**6**_**O**_**3**_: C, 62.84; H, 5.75; N, 19.99. Found: C, 63.09; H, 5.67; N, 20.17.

*4-((4-Chlorophenyl)diazenyl)-5-methyl-2-(phenyl(piperidin-1-yl)methyl)-2,4-dihydro-3H-pyrazol-3-one (4c)* Yellow crystals; 60% yield; m.p (170–172)°C; IR (ν/cm^−1^): 3438.5 (OH); ^1^H-NMR (500 MHz, DMSO-d_6_): δ 1.44–1.57 (6H, m), 2.16 (3H, s), 2.51–2.52 (4H, m), 5.93 (1H, s), 7.24–7.26 (2H, m, Ar–H), 7.32–7.45 (4H, m, Ar–H), 7.54–7.70 (3H, m, Ar–H), 11.56 (1H, s, OH_exchange with D2O_). ^13^C-NMR (125.6 MHz, DMSO-d_6_): 12.24, 23.37, 26.33, 44.73, 50.54, 74.42, 118.03, 118.70, 128.14, 128.67, 129.70, 129.84, 130.03, 135.13, 193.80. EI-MS (m/z, %): 409.62 [M^+2^]. Calc. for **C**_**22**_**H**_**24**_**ClN**_**5**_**O**: C, 64.46; H, 5.90; N, 17.09. Found: C, 64.62; H, 6.04; N, 17.31.

#### Synthesis of compound 5, 6 (a-b)

These compounds were synthesized through the reaction of equimolar amounts of **2a** or **2b**, benzaldehyde, and aniline or 4-aminoantipyrine (5mmol), following the procedure defined above for compounds **3,4 (a-c)**. The product was recrystallized from ethanol.

*3-Methyl-1-(phenyl(phenylamino)methyl)-4-(phenyldiazenyl)-1H-pyrazol-5-ol (5a)* Yellow crystals; 54% yield; m.p (186–188)°C; IR (ν/cm^−1^): 3307.7(NH), 3438.5 (OH); ^1^H-NMR (500 MHz, DMSO-d_6_): δ 2.13 (3H, s, CH_3_), 6.60 (1H, s, Ar–CH*-NH), 6.62- 6.91 (2H, m, Ar–H), 7.08–7.33 (4H, m, Ar–H), 7.35–7.43 (4H, m, Ar–H), 7.48–7.55 (5H, m, Ar–H), 7.56 (1H, s, NH_exchange with D2O_), 11.54 (1H, s, OH_exchange with D2O_). ^13^C-NMR (125.6 MHz, DMSO-d_6_): 12.11, 63.91, 113.84, 114.38, 116.13, 121.49, 125.58, 127.48, 128.69, 128.94, 129.19, 129.33, 129.72, 130.10, 141.94, 147.35, 160.74. EI-MS (m/z, %): 383.46 [M^+^]. Calc. for **C**_**23**_**H**_**21**_**N**_**5**_**O**: C, 72.04; H, 5.52; N, 18.26. Found: C, 71.93; H, 5.64; N, 18.43.

*3-Methyl-1-((2-nitrophenylamino)(phenyl)methyl)-4-(phenyldiazenyl)-1H-pyrazol-5-ol(5b)* Yellow crystals; 56% yield; m.p (250–252)°C; IR (ν/cm^−1^): 3250.3 (NH), 3438.5 (OH); ^1^H-NMR (500 MHz, DMSO-d_6_): δ 2.18 (3H, s, CH_3_), 6.64 (1H, s, Ar–CH*-NH), 6.76- 6.78 (1H, d, ^*3*^* J* = 7.8 Hz, Ar–H), 6.85- 6.86 (1H, m, Ar–H), 7.87–7.90 (1H, m, Ar–H), 7.95–6.97 (1H, m, Ar–H), 7.10–7.13 (3H, m, Ar–H), 7.31–7.33 (1H, m, Ar–H), 7.34–7.35 (1H, t, ^*3*^* J* = 8 Hz, Ar–H), 7.37–7.40 (1H, m, Ar–H), 7.43–7.84 (4H, m, Ar–H), 11.79 (1H, s, NH_exchange with D2O_), 14.38 (1H, s, OH_exchange with D2O_). ^13^C-NMR (125.6 MHz, DMSO-d_6_): 11.84, 114.87, 116.84, 117.16, 124.64, 126.31, 129.45, 129.72, 130.06, 132.72, 134.83, 135.37, 137.06, 137.89, 148.24, 159.66. EI-MS (m/z, %): 428.72 [M^+^]. Calc. for **C**_**23**_**H**_**20**_**N**_**6**_**O**_**3**_: C, 64.48; H, 4.71; N, 19.62. Found: C, 64.65; H, 4.82; N, 19.89.

*4-(((5-Hydroxy-3-methyl-4-(phenyldiazenyl)-1H-pyrazol-1-yl)(phenyl)methyl) amino)-1,5-dimethyl-2-phenyl-1,2-dihydro-3H-pyrazol-3-one (6a)* Yellow crystals; 45% yield; m.p (202–204)°C; IR (ν/cm^−1^):1646.3(CO), 3195.3 (NH), 3410.3 (OH); ^1^H-NMR (500 MHz, DMSO-d_6_): δ 2.17 (3H, s, CH_3_*), 2.40 (3H, s, CH_3_*), 2.81 (3H, s, N(CH_3_*)), 6.25 (1H, s, Ar–CH*-NH) 7.31- 7.35 (6H, m, Ar–H), 7.36–7.50 (6H, m, Ar–H), 7.86–7.88 (1H, t, ^*3*^* J* = 7.9 Hz, Ar–H), 7.88–7.89 (2H, d, ^*3*^* J* = 7.7 Hz, Ar–H) 7.97 (1H, s, NH_exchange with D2O_), 9.42 (1H, s, OH_exchange with D2O_). ^13^C-NMR (125.6 MHz, DMSO-d_6_): 11.74, 12.84, 29.04, 69.44, 110.73, 121.85, 123.80, 127.08, 127.46, 128.63, 128.91, 129.33, 129.39, 130.42, 131.63, 135.77, 135.96, 140.74, 141.99, 147.61, 151.21, 163,20. EI-MS (m/z, %): 493.30 [M^+^]. Calc. for **C**_**28**_**H**_**27**_**N**_**7**_**O**_**2**_**:** C, 68.14; H, 5.51; N, 19.87. Found: C, 68.38; H, 5.63; N, 20.15.

*4-(((5-Hydroxy-3-methyl-4-((2-nitrophenyl)diazenyl)-1H-pyrazol-1-yl)(phenyl) methyl)amino)-1,5-dimethyl-2-phenyl-1,2-dihydro-3H-pyrazol-3-one (6b)* orange crystals; 55% yield; m.p (230–232)°C; IR (ν/cm^−1^):1671.5(CO), 3150.7(NH), 3338.9 (OH); ^1^H-NMR (500 MHz, DMSO-d_6_): δ 2.17 (3H, s, CH_3_), 2.40 3H, s, CH_3_*), 2.81 (3H, s, N(CH_3_*), 6.24 (1H, s, Ar–CH*-NH), 7.31- 7.36 (6H, m, Ar–H), 7.45–7.51 (4H, m, Ar–H), 7.62–7.65 (1H, m, Ar–H), 7.84–7.84 (1H, d, ^*4*^* J* = 1.3 Hz, Ar–H), 7.92–7.92 (1H, d, ^*4*^* J* = 1.3 Hz, Ar–H), 7.96–7.97 (1H, d, ^*3*^* J* = 7.9 Hz, Ar–H), 7.76 (1H, t, ^*3*^* J* = 8 Hz, Ar–H), 7.86 (1H, t, ^*3*^* J* = 7.7 Hz, Ar–H), 8.02 (1H, d, ^*3*^* J* = 7.6 Hz, Ar–H), 8.17 (1H, d, ^*3*^* J* = 6 Hz, Ar–H), 9.12 (1H, s, Ar–CH*-NH), 8.17 (1H, s, NH_exchange with D2O_), 9.37 (1H, s, OH_exchange with D2O_). ^13^C-NMR (125.6 MHz, DMSO-d_6_): 11.74, 12.91, 26.39, 69.44, 110.73, 123.80, 123.95, 124.80, 127.06, 127.46, 128.63, 128.91, 129.39, 130.84, 131.27, 134.34, 135.77, 135.96, 140.74, 142.08, 144.06, 144.28, 147.57, 163.20. EI-MS (m/z, %): 538.59 [M^+^]. Calc. for **C**_**28**_**H**_**26**_**N**_**8**_**O**_**4**_: C, 62.44; H, 4.87; N, 20.81. Found: C, 62.17; H, 5.04; N, 21.07.

### Biological activity

#### Cytotoxicity

The RPMI-1640 complete medium L-Glutamine (Lonza Verviers SPRL, Belgium, cat#12-604F) was used to cultivate the liver cancer (HepG2), normal liver (THLE2) cell lines purchased from the National Cancer Institute, Egypt. The cell lines were supplemented with 10% fetal bovine serum (Sigma-Aldrich, MO, USA) and 1% penicillin–streptomycin. Compounds were added to the cells on the second day at doses of “0, 0.01, 0.1, 1, 10, and 100 μM”. Cell viability was assessed after 48 h using the MTT solution (Promega, USA) [38, 39].

#### EGFR and CDK-2 kinase inhibitory assay

VEGFR2 (KDR) kinase assay kit “BPS Bioscience, Corporation catalog # 40,325” and CDK-2 luminescence kinase Assay kit “Catalog #79,599, Kinase-Glo Plus, Promega, USA” were performed. The inhibitory efficacy of compounds **4a, 5a,** and **6b** against VEGFR2 and CDK-2 was determined using kinase inhibitory assays. The following formula was used to determine the proportion of autophosphorylation inhibition by compounds: $100-[\frac{A control}{A treated}-Control)]$ [40].

#### Investigation of apoptosis

##### Annexin V/PI staining and cell cycle analysis

HepG2 cells (3–5 105 cells/well) were cultured onto 6-well culture plates and incubated overnight. After that, cells were treated for 48 h with compounds **5a** and **5b** at the IC_50_ concentrations. The cells and medium supernatants were then washed with ice-cold PBS. After that, 100 mL of annexin binding buffer solution was added to the cell suspension "25 mM CaCl2, 1.4 M NaCl, and 0.1 M Hepes/NaOH, pH 7.4″ and incubation with “Annexin V-FITC solution (1:100) and propidium iodide (PI)” at a concentration equals 10 µg/mL in the dark for 30 min. The Cytoflex FACS system was then used to acquire the stained cells. cytExpert was used for data analysis [41–43].

### In Silico studies

#### Molecular docking

Ligands were constructed and energy-minimized at AMBER partial charges and modified forcefield using ChemDraw. Cyclin-dependent kinase (CDK-2; PDB = 2a4l), and Vascular epidermal growth factor receptor (VEGFR2; PDB = 3WZE) were deposited and structurally prepared. Then, molecular docking was performed using AutoDock Vina 1.2.0 software suit (Scripps Research, La Jolla, CA, United States) and Chimera-UCSF was used for visualization and binding interaction analysis [38–40]

#### ADME pharmacokinetics

The synthesized compounds were assessed for their chemo-informatics properties using the Lipinski rule of five (RO5). Multiple online servers such as Molinspiration (http://www.molin spiration.com/) and Molsoft (http:// www.molsoft.com/) were employed to predict the molecular properties designed compounds [38, 39, 44, 45].

#### Statistical analysis

Data were statistically analyzed using unpaired student t-test or one-way ANOVA (GraphPad Prism software version 8 for Windows). Data were expressed as Mean ± standard deviation, and results were considered statistically significant when p ≤ 0.05.

## Results and discussion

### Chemistry

**Scheme 1.**
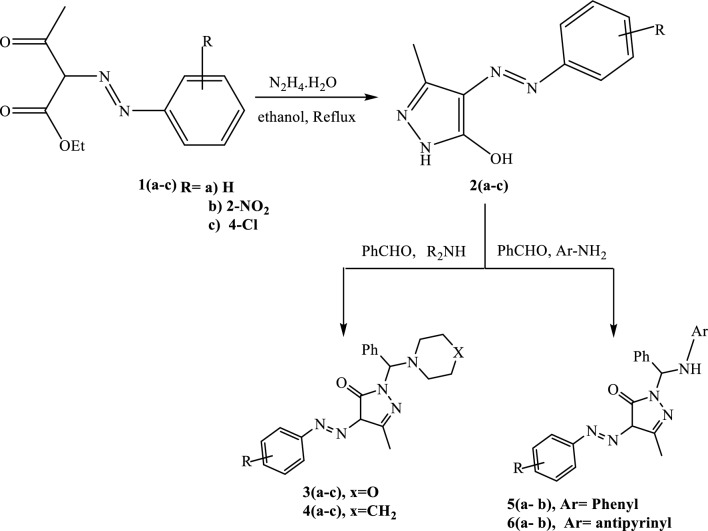
Synthesis of *N*-Mannich bases of pyrazole derivatives

The classic route to *N-*Mannich bases of 3-methyl-4-aryl azo pyrazol-5-ol **2 (a-c)** and associated compounds include their reactions with benzaldehyde and the appropriate amines. Benzaldehyde is considered the main component in this reaction. This reaction has been accomplished by treating **2 (a-c)** with benzaldehyde and cyclic secondary amines (morpholine or piperidine) to afford **3 (a-c)**, **4 (a-c)**, respectively (Scheme 1). The chemical structures of **3 (a-c)** and **4 (a-c)** are consistent with their analytical and spectral data. The formed products were characterized by TLC. The IR spectra of these newly synthesized compounds showed strong absorption broad bands around 3434–3430 (OH), and 1220–1215 cm^−1^ (C-N stretch of *sec*-heterocyclic amine). In addition, the disappearance of stretching bands at 3300, 2820, and 2720 for NH and CHO evidence the formation of the structure. The ^1^H-NMR spectrum of compound **3a** indicated the presence of a singlet for (Ph-CH*) at δ = 5.90 and two multiples at 3.55- 3.61 (CH_2_─O─CH_2_) and 2.51- 2.54 ppm (CH_2_─N─CH_2_), and the presence of a singlet for (OH exchange with D_2_O) at δ = 12.96. The mass spectra of **3a** and **4a** indicated the molecular ion peaks at *m/z* 377.61 and 374.92, respectively. Moreover, compound **3c** indicated the molecular ion peak M^+^ and the molecular ion isotopic peak M^+2^ at *m/z* 410 and 412, respectively, evidence of the presence of chloride (Cl^−^). The ^13^C-NMR spectra showed signals for phenyl, piperidinyl, morpholino, and pyrazolone rings at the expected regions. Regarding **5,6 (a, b)**, the synthesis was achieved through the reaction of **2 (a-c)** with aldimines derived from benzaldehyde and primary aromatic or heterocyclic amines which resulted in the synthesize of 1-(arylaminobenzyl)-4-arylazopyrazol-5-ol **5 (a, b)** and **6 (a, b)**. The reaction depends on the alteration in the amine component of the aminobenzyl moiety of the new compounds. The chemical structures of **5,6 (a, b)** were confirmed based on spectral and analytical data. The IR spectra of the newly synthesized compounds revealed strong absorption bands around 3425–3420 (OH), 3270 -3266 (NH) for **5,6 (a, b),** and 1725–1722 (CO) for **5 (a, b)**. In addition, the presence of stretching bands at 1332–1225 cm^−1^ (C─N stretch of *sec*-aromatic amine) for **5 (a, b)**. The disappearance of the stretching bands at 2822 and 2721 (2 peaks) for CHO as well as, the vanishing of the primary NH_2_ group and the appearance of secondary NH between 3341- 3337 represent evidence for the formation of the structure. The ^1^H-NMR spectra are an additional proof for the synthesize of **6a** which showed the presence of a singlet for (Ph-CH*-NH) at δ = 6.25, two additional singlet peaks at 2.40, 2.81 for (C-CH_3_) and (N-CH_3_). The presence of two singlets for (NH and OH exchange with D_2_O) at δ = 7.97 and 9.42, respectively. Additionally, the aromatic protons (15 H) split into two multiples at 7.31- 7.35 and 7.36–7.50 ppm for (12 H), beside one triplet at 7.86–7.88 with a coupling constant (*J* = 7.9 Hz) (1H) and one doublet at 7.88–7.89 ppm with a coupling constant (*J* = 7.7 Hz) (2H). The ^13^C-NMR spectrum of **6a** also confirmed the expected structure by giving carbon signals at δ163.2 for C = O, and two peaks at δ 12.84, 29.04 for CH_3_ and N-CH_3_. Respectively. Finally, the mass spectra of **6a** revealed the molecular ion peaks at *m/z* 493.2. All spectra for characterization including NMR, mass were completely supported in the supplementary file (S1-S10), and elemental analyses report, Figure S11 and Table S1.

### Biology

#### Cytotoxicity

Using the MTT assay, the cytotoxicity of the synthesized compounds against HepG2 cells was screened. As seen in Table 1, compounds **4a, 5a,** and **6b** showed potent cytotoxicity against HepG2 with IC_50_ values of 4.4, 3.46 and 2.52 µM compared to doxorubicin (IC_50_ = 2.051 µM) and Roscovitine (IC_50_ = 1.9 µM). While compounds **3c**, **4b**, **5b,** and **6a** showed moderate cytotoxicity against HepG2 cancer cells with IC_50_ values of 5.29, 9.34, 17.09, and 17.31 µM. Furthermore, they weren’t cytotoxic against the THLE2 cells with higher IC_50_ values. Additionally, compounds **4b** and **6a** showed encouraging cytotoxicity against HepG2 cells with IC_50_ values of 9.33 and 5.26 μM, respectively. Other compounds exhibited weak cytotoxic activities. Dose–response curves were provided in the Supplementary file (Figure S12 and S13).

**Table 1:** Cytotoxic IC_50_ values of the tested compounds against HepG2 and THLE2 cell lines using the MTT assay

Compounds	IC_50_ ± SD* (µM)	
	HepG2	THLE2
3a	33.86 ± 1.89	41.22 ± 1.19
3b	55.47 ± 3.1	47.21 ± 1.85
3c	17.09 ± 0.96	34.5 ± 2.1
4a	4.43 ± 0.25	27.31 ± 1.15
4b	9.34 ± 0.52	72.4 ± 2.15
4c	25.21 ± 1.41	34.7 ± 1.05
5a	3.46 ± 0.16	48.67 ± 2.05
5b	17.31 ± 0.8	65.2 ± 2.3
6a	5.27 ± 0.24	75.3 ± 3.3
6b	2.52 ± 0.12	60.16 ± 2.54
Sorafenib	2.05 ± 0.09	16.98 ± 0.72
Roscovitine	4.18 ± 0.23	23.6 ± 0.98

NT: Not Tested. Dose–response curves were provided in the Supplementary file (Figure S12 and S13)

^*^Values are expressed as Mean ± SD of three independent triplets (n = 3)

#### EGFR and CDK-2 kinase inhibitory assay

To determine the effective molecular target of compounds **4b**, **5a**, and **6b**, which exhibited the highest cytotoxic activity against HepG2 cells, we tested them against the VEGFR2/CDK-2 inhibitory activities. As seen in Table 2, compound **5a** exhibited promising dual VEGFR2/CDK-2 inhibition activities, it had IC_50_ value of 0.267 μM with VEGFR2 inhibition of 91.7% compared to Sorafenib (IC_50_ = 0.03 μM, 95.4%) and it had IC_50_ value of 0.311 μM with CDK-2 inhibition of 91% compared to Roscovitine (IC_50_ = 0.556 μM, 93.4%).

**Table 2:** IC_50_ values of EGFR and CDK-2 kinase activities of the tested compounds

Compound	VEGFR2 kinase		CDK-2 kinase	
	IC_50_ [μM]*	% of EGFR inhibition [10 µM]	IC_50_ [μM]*	% of CDK-2 inhibition at[10 µM]
4a	0.55 ± 0.006	90.5 ± 2.1	0.205 ± 0.011	90.9 ± 1.9
5a	0.267 ± 0.001	91.7 ± 3.1	0.311 ± 0.011	91 ± 2.1
6b	0.2 ± 0.001	93.2 ± 2.9	0.458 ± 0.017	88.7 ± 2.6
Sorafenib	0.03 ± 0.002	95.4 ± 2.7	–	–
Roscovitine			0.556 ± 0.001	92.1 ± 2.7

“IC_50_ values were calculated using sigmoidal non-linear regression curve fit of percentage inhibition against five concentrations of each compound”

^*^Values are expressed as an average of three independent replicates

Additionally, compound **4a** exhibited promising dual VEGFR2/CDK-2 inhibition activities; it had an IC_50_ value of 0.55 μM with VEGFR2 inhibition of 90.5%, and it had an IC_50_ value of 0.205 μM with CDK-2 inhibition of 90.9%. In contrast, compound **6b** exhibited promising dual VEGFR2/CDK-2 inhibition activities; it had an IC_50_ value of 0.2 μM with VEGFR2 inhibition of 93.2%, and it had an IC_50_ value of 0.458 μM with CDK-2 inhibition of 88.7%.

#### Apoptotic investigation

##### Annexin V/PI staining with cell cycle analysis

**Fig. 5:**
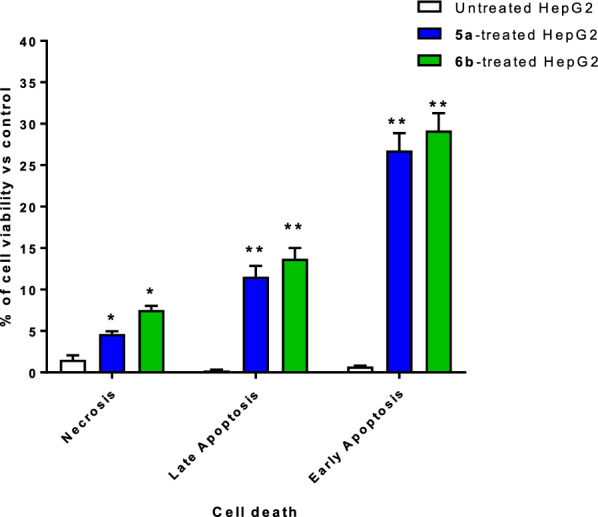
Apoptosis/necrosis assessment using Annexin-V/Propidium Iodide staining of untreated and **5a** and **6b-**treated HepG2 cells at the IC_50_ values, 48 h. “*(P ≤ 0.05) and **(P ≤ 0.001) significantly different between untreated and treated cells”

Flow cytometric examination of Annexin V/PI staining of untreated and treated HepG2 cells was used to investigate the apoptotic activity of compounds **5a** and **6b**. Figure 5 demonstrated that compounds **5a** and **6b** considerably induced cell death through apoptosis, leading to an increase in the overall apoptotic cell death by 38.32% and 42.9%, respectively, compared to the untreated control group (0.95%). Additionally, they induced necrosis by 4.63% and 7.54% compared to 1.52% in untreated control. Hence, compound treatments induced apoptotic and necrotic cell death as a dual cell death mechanism. Annexin V/PI staining histograms are supported in the Supplementary (Figure S14).

**Fig. 6:**
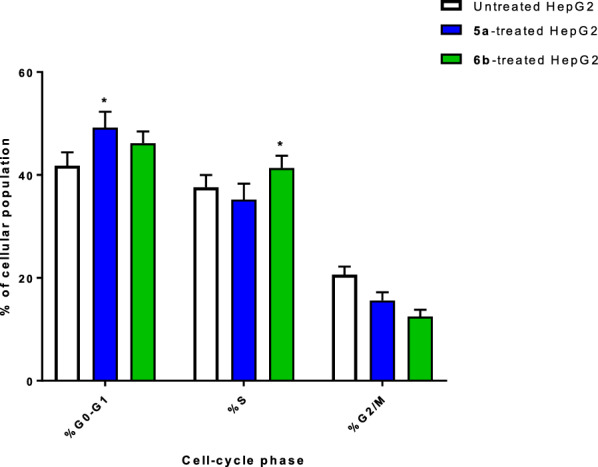
Percentage of cell population at each cell cycle “G1, S, and G2/M” in untreated and **5a** and **6b-**treated HepG2 cells with the IC_50_ values, 48 h using DNA content-flow cytometry aided cell cycle analysis. “*(P ≤ 0.05) significantly different between untreated and treated cells”

After exposing the cells to a cytotoxic chemical, DNA flow cytometry was utilized to count how many were in each cell phase. As can be shown in Fig. 6, the cell population in the G0-G1-phase was considerably raised by 49.18% after treatment with compound **5a**, compared to the control 41.8%, whereas the cell population in the S-phase was increased by 41.33% after treatment with compound **6b** compared to the control 37.59%. Hence, compounds **5a** and **6b** halted the cell proliferation at G0-G1 and S-phases, respectively. Cell cycle analysis histograms are supported in the Supplementary (Figure S14).

**Fig. 7:**
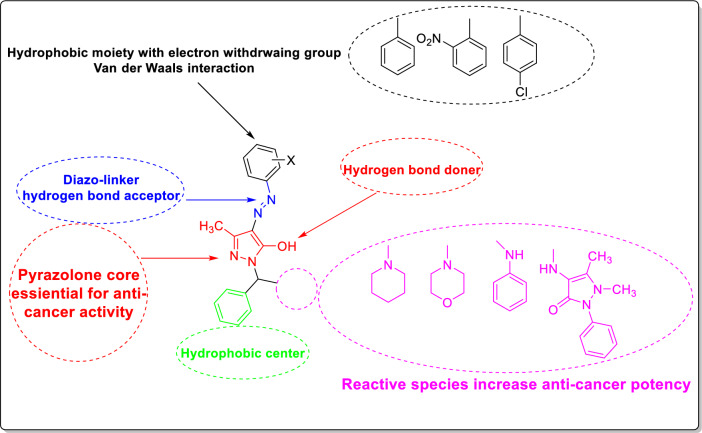
A proposed structure–activity relationship (SAR) model for the activity of the synthesized derivatives. Reactive moieties were added to the central scaffold (pyrazolone) with high cytotoxic potencies

Depending on the structure and the cytotoxicity of tested compounds, structure–activity relationship (SAR) model can be generated as summarized in Fig. 7.

In agreement with previous studies [27, 46–48] regarding the investigation of the anti-cancer activity of pyrazole-based derivatives through cytotoxicity, enzyme targeting, and mechanism of cell death, it was previously agreed that pyrazole derivatives exhibited potent cytotoxicity against a panel of cancer cells including liver cancer, induced potent CDK-2 and VEGFR2 inhibition that led to induction of apoptosis as the effective of cell death. As a result, pyrazole derivatives have great promise as a future generation of targeted cancer chemotherapeutics.

### Molecular modeling

One well-known kinase involved in breast cancer progression is vascular endothelial growth factor receptor-2 (VEGFR2) kinase. As a result of its importance in apoptosis and widespread overexpression in tumors, inhibiting VEGFR2 has emerged as a potential therapeutic target for numerous cancers that rely on this cell death pathway [41, 42]. Regarding the pharmacophoric regions of VEGFR2, it contains a heteroaromatic ring, H-bond donor/acceptor and a lipophilic tail [49–54], and the tested compounds’ structures contain these regions.

In mammalian cells, cyclin-dependent kinases control the progression through the cell cycle (CDKs). Proteins necessary for DNA replication and cell division are phosphorylated by these serine/threonine kinases, which regulate the cell cycle. Hence, CDK inhibitors can induce growth arrest and apoptosis in cancer cells [43].

**Fig. 8:**
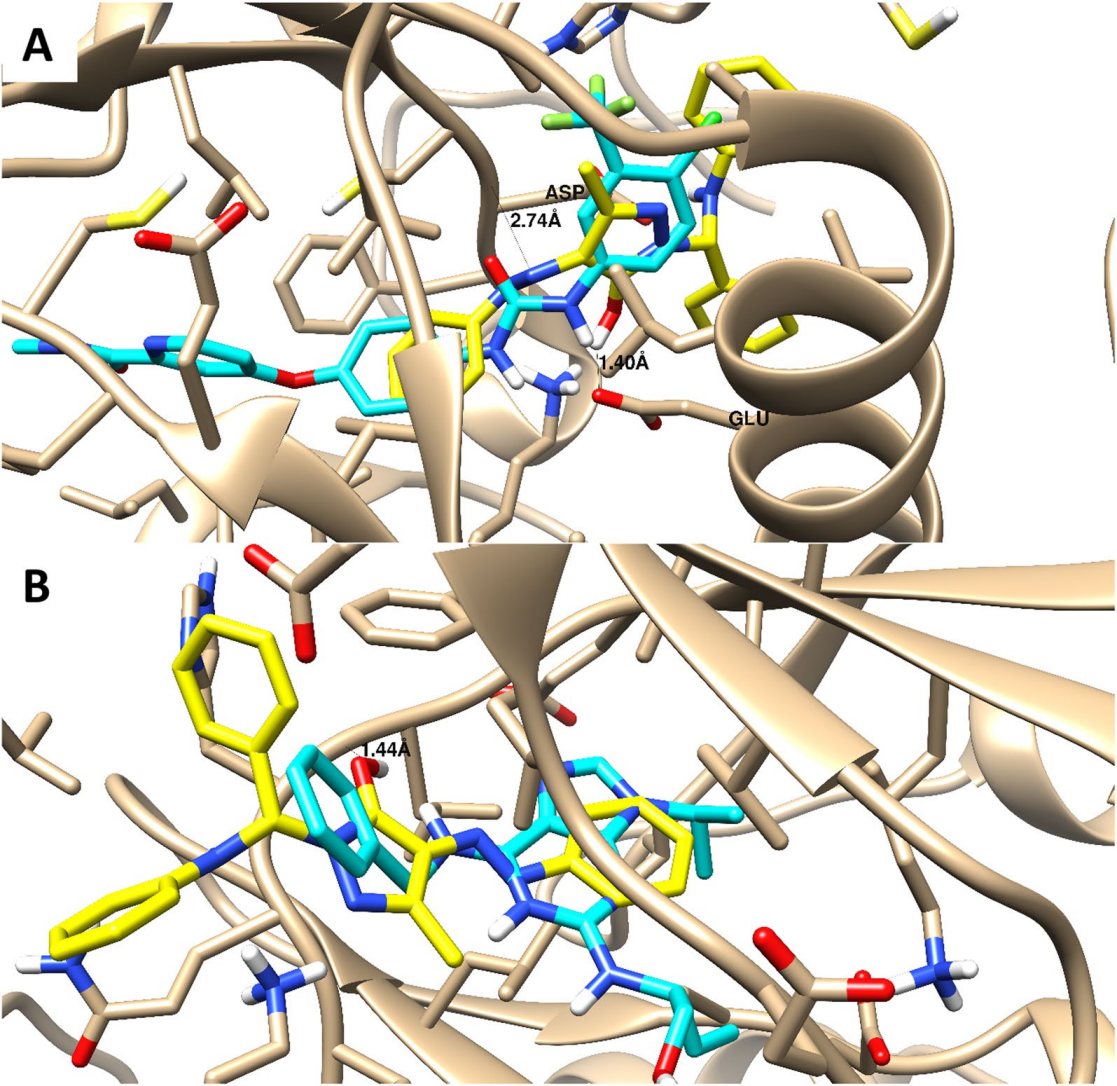
Binding disposition and interactive mode of the docked compound (**6b** Yellow-colored) in comparison with the co-crystallized ligands of both proteins (Cyan-colored) VEGFR2 (**A**) and CDK-2 (**B**). 3D images were generated by Chimera-UCSF

A molecular docking study was carried out to highlight the binding affinity of the tested compounds towards VEGFR2 and CDK-2 proteins. As summarized in Table 3, compounds **4a, 5a,** and **6b** exhibited good binding interactions against VEGFR2 with binding energies of -13.59 to -23.86 kcal/mol and formed hydrogen bond interactions with Asp 1046 as a critical interactive amino acid. Additionally, compounds exhibited good binding interactions against CDK-2 with binding energies of -7.9 to -10.75 kcal/mol and formed arene-cation or hydrogen bond interactions with Lys 89. The Molecular docking study exhibited the dual VEGFR2/CDK-2 target inhibition for the tested compounds, especially for compound **6b,** due to the highest binding energy and maintaining the same binding mode of the co-crystallized ligands. It made two H-bonds with Asp 1046 and one H-bond with Lys 868 through (-N = N-), -OH to pyrazole, and the amide linker inside the VEGFR2 protein. Besides, it made one H-bonds and Van der Waals force with Lys 89 as (ion-induced dipole) through the pyrazole moiety inside the binding site of CDK-2 protein, as seen in Fig. 8.

**Table 3:** Summary of ligand-receptor interactions with binding energy of the docked compounds **(4a, 5a, 6b)** towards VEGFR2 and CDK-2 proteins*

	VEGFR2		CDK-2	
	Binding energy (Kcal/mol)	Ligand-receptor interactions	Binding energy (Kcal/mol)	Ligand-receptor interactions
Co-ligand^#^	− 12.91	1 H-bond with Asp 1046	− 11.2	Arene-cation with Lys 89
4a	− 13.59	2HB with Glu 885, Asp 1046 Arene-cation with Lys 868	− 7.9	2 arene-cation with Lys 89
5a	− 18.78	2 HB with Glu 885, and Asp 1046 Arene-cation with Lys 868	− 16.60	1 H-bond and arene-cation with Lys 89
6b	− 23.86	1HB with Asp 1046 1 HB with Glu 885	− 10.75	1 H-bond and arene-cation with Lys 89

Docking calculations were validated using RMSD calculation value of 0.7 through the self-docking

^*^Docking calculation was carried out using AutoDock Vina and visualization was made by Chimera-UCSF software

^#^Co-lig is the co-crystallized ligand inside the binding sites of two proteins

### Physicochemical and pharmacokinetic properties

Compounds **4a, 5a**, and **6b** were investigated for their physicochemical and drug-likeness properties. As seen in Table 4, the tested compounds showed promising values according to Lipinski’s rule of five of "molecular weight, number of rotatable bonds, H-bond donor, and acceptors along with a number of violations [40, 42].

**Table 4:** Molecular properties of and drug-likeness

Comp	Molsoft										SwissADME
	HBA	HBD	Solubility (mg/L)	DrugScore	MWt (D)	MV (A^3^)	PSA (A^2^)	Log p	Number of stereo centers	nviolations	Drug likeness (Lipinski Pfizer filter)
4a	5	1	23.91	0.67	375.21	375.93	51.60	4.35	1	0	Yes
5a	4	2	11.02	− 0.33	383.17	356.66	58.74	4.99	1	0	Yes
6b	7	2	134.38	0.77	538.21	519.09	116.38	2.87	1	1	Yes

“Mwt: Molecular Weight, MV: Molecular Volume, PAS: Polar Surface Area, Log p: Log P: Octanol–water partition coefficient, nrotb: number of rotatable bonds, nviolations: number of violations, HBA: Hydrogen Bond Acceptor, HBD: Hydrogen Bond Donor, drug-likeness score, compounds having negative or zero value should not be considered as a drug like”. Drug likeness (Lipinski Pfizer filter) / “Yes, drug-like” MW ≤ 500, Log p ≤ 4.25, HBA ≤ 10 and HBD ≤ 5”

## Conclusion

New series *N*-Mannich bases of Azo pyrazole derivatives **3,4 (a-c)** and **5,6 (a, b)** were synthesized by treating **2 (a-c)** with benzaldehyde and the appropriate primary and secondary amines. The use of morpholine and piperidine as the sec amine component in the Mannich reaction and aniline and 4-aminoantipyrine as the primary amine with azo pyrazole derivatives **2 (a-c)** led to the formation of a new series of *N*-Mannich base pyrazole derivatives. Compound **6b** showed potent cytotoxicity against HepG2 compared to doxorubicin and Roscovitine. Furthermore, it was safe against the THLE2 cells with a much higher IC_50_ value. Compound **6b** exhibited promising dual VEGFR2/CDK-2 inhibition activities. It dramatically increased apoptosis in HepG2 cancer cells, increasing total cell population in apoptosis compared to the control group. In addition, compound **6b** halted the cell population at the S phase. Thus, by inhibiting VEGFR2/CDK-2, compound 6b has the potential to be a powerful drug against liver cancer.

## Supplementary Information

Supplementary material 1.

## Data Availability

The data that support the findings of this study are available in the supplementary material of this article.

## Abbreviations

VEGFR2: Vascular endothelial growth factor receptor 2
CDK-2: Cyclin-dependent kinase 2
IC_50_: Half-maximal inhibitory concentration
HePG2: Liver cancer cells
THLE2: Normal liver cells
HCC: Hepatocellular carcinoma
G2/M, S, G1, G0: Cell cycle phases
