# Supplementary material for: Design, synthesis, and biological investigations of new pyrazole derivatives as VEGFR2/CDK-2 inhibitors targeting liver cancer
Source: BMC Chem. 2024 Oct 24;18(1):208. doi: 10.1186/s13065-024-01314-z (PMC11520136; doi:10.1186/s13065-024-01314-z)
Supplement: Supplementary file 1 — Supplementary material 1. [file 13065_2024_1314_MOESM1_ESM.docx]

**Design, Synthesis, and Biological Investigations of New Pyrazole Derivatives as VEGFR2/CDK-2 Inhibitors Targeting Liver Cancer.**

Manar G. Salem ^a *^, Mohamed S. Nafie ^b,c^, Aya A. Elzamek ^a^, Hosam A. Elshihawy ^a^, Mamdouh A. Sofan ^d^, Elham Negm ^d^.

a *Pharmaceutical Organic Chemistry Department, Faculty of Pharmacy, Suez Canal University, P.O. 41522 Ismailia, Egypt*

*b* *Department of Chemistry, College of Sciences, University of Sharjah, Sharjah (P.O. 27272), United Arab Emirates (UAE).*

*c Chemistry Department, Faculty of Science, Suez Canal University, Ismailia (P.O 41522), Egypt*

*d Department of Chemistry, Faculty of Science, Damietta University, New Damietta, Egypt.*

*Corresponding author:

**Manar G. Salem:** Email: manar.galal@pharm.suez.edu.eg

# Table of Contents

| **Title** | **Page** |
| --- | --- |
| Compound **3a** (chemical structure-general formula-molecular weight) | 3 |
| Figure S1 a: 1H NMR spectrum of compound **3a** | 4 |
| Figure S1 b: ^13^C-NMR spectrum of compound **3a** | 5 |
| Figure S1 c: Mass spectrum of compound **3a** | 6 |
| Compound **3b** (chemical structure-general formula-molecular weight) | 7 |
| Figure S2 a: 1H NMR spectrum of compound **3b** | 8 |
| Figure S2 b: ^13^C-NMR spectrum of compound **3b** | 9 |
| Figure S2 c: Mass spectrum of compound **3b** | 10 |
| Compound **3c** (chemical structure-general formula-molecular weight) | 11 |
| Figure S3 a: 1H NMR spectrum of compound **3b** | 12 |
| Figure S3 b: ^13^C-NMR spectrum of compound **3b** | 13 |
| Figure S3 c: Mass spectrum of compound **3b** | 14 |
| Compound **4a** (chemical structure-general formula-molecular weight) | 15 |
| Figure S4 a: 1H NMR spectrum of compound **4a** | 16 |
| Figure S4 b: ^13^C-NMR spectrum of compound **4a** | 17 |
| Figure S4 c: Mass spectrum of compound **4a** | 18 |
| Compound **4b** (chemical structure-general formula-molecular weight) | 19 |
| Figure S5 a: 1H NMR spectrum of compound **4b** | 20 |
| Figure S5 b: ^13^C-NMR spectrum of compound **4b** | 21 |
| Figure S5 c: Mass spectrum of compound **4b** | 22 |
| Compound **4c** (chemical structure-general formula-molecular weight) | 23 |
| Figure S6 a: 1H NMR spectrum of compound **4c** | 24 |
| Figure S6 b: ^13^C-NMR spectrum of compound **4c** | 25 |
| Figure S6 c: Mass spectrum of compound **4c** | 26 |
| Compound **5a** (chemical structure-general formula-molecular weight) | 27 |
| Figure S7 a: 1H NMR spectrum of compound **5a** | 28 |
| Figure S7 b: ^13^C-NMR spectrum of compound **5a** | 29 |
| Figure S7 c: Mass spectrum of compound **5a** | 30 |
| Compound **5b** (chemical structure-general formula-molecular weight) | 31 |
| Figure S8 a: 1H NMR spectrum of compound **5b** | 32 |
| Figure S8 b: ^13^C-NMR spectrum of compound **5b** | 33 |
| Figure S8 c: Mass spectrum of compound **5b** | 34 |
| Compound **6a** (chemical structure-general formula-molecular weight) | 35 |
| Figure S9 a: 1H NMR spectrum of compound **6a** | 36 |
| Figure S9 b: ^13^C-NMR spectrum of compound **6a** | 37 |
| Figure S9 c: Mass spectrum of compound **6a** | 38 |
| Compound **6b** (chemical structure-general formula-molecular weight) | 39 |
| Figure S10 a: 1H NMR spectrum of compound **6b** | 40 |
| Figure S10 b: ^13^C-NMR spectrum of compound **6b** | 41 |
| Figure S10 c: Mass spectrum of compound **6b** | 42 |
| Figure S11: Elemental analysis values | 43 |
| Table S1: Elemental analysis codes | 44 |
| **Supplementary part for biology work** | 46 |
| Figure S12: Dose-response curve for the cytotoxicity of tested compounds (10 compounds) against HepG2 cancer cells. | 46 |
| Figure S13: Dose-response curve for the cytotoxicity of tested compounds (**10** compounds) against THLE2 normal liver cells. | 47 |
| Figure S14: Raw data of flow cytometry analysis; Annexin V/PI staining and Cell cycle analysis of compounds **5a** (s31) and **6b** (s20). | 48-52 |

# Compound 3a

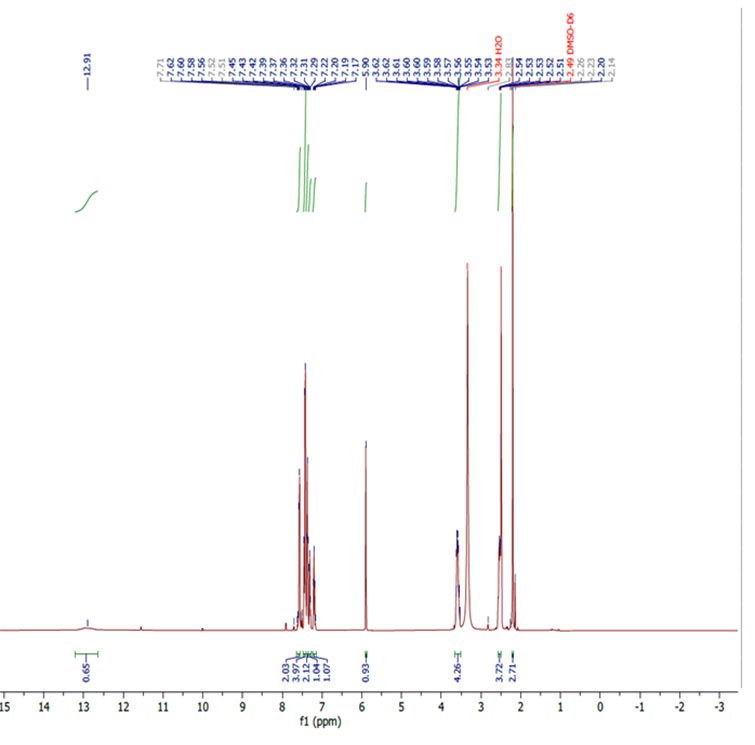


Figure S1 a: ^1^H NMR spectrum of compound **3a**

**
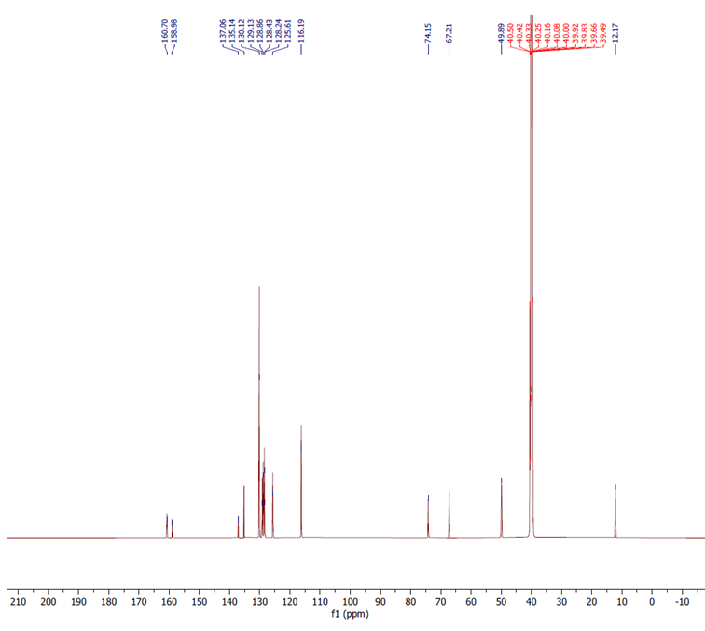
**

Figure S1 b: ^13^C-NMR spectrum of compound **3a**


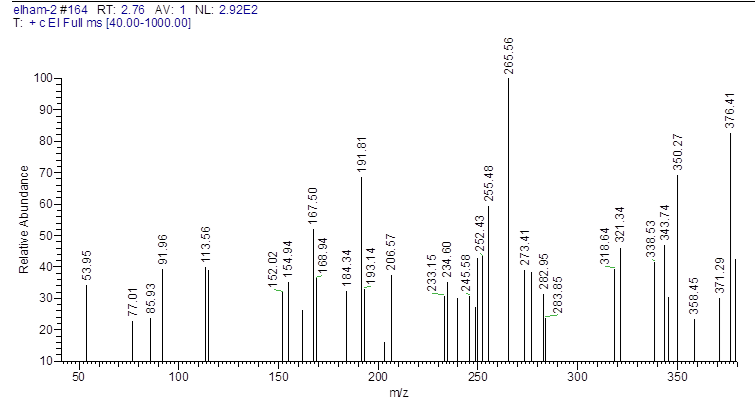


Figure S1 c: Mass spectrum of compound **3a**

**Compound 3b**

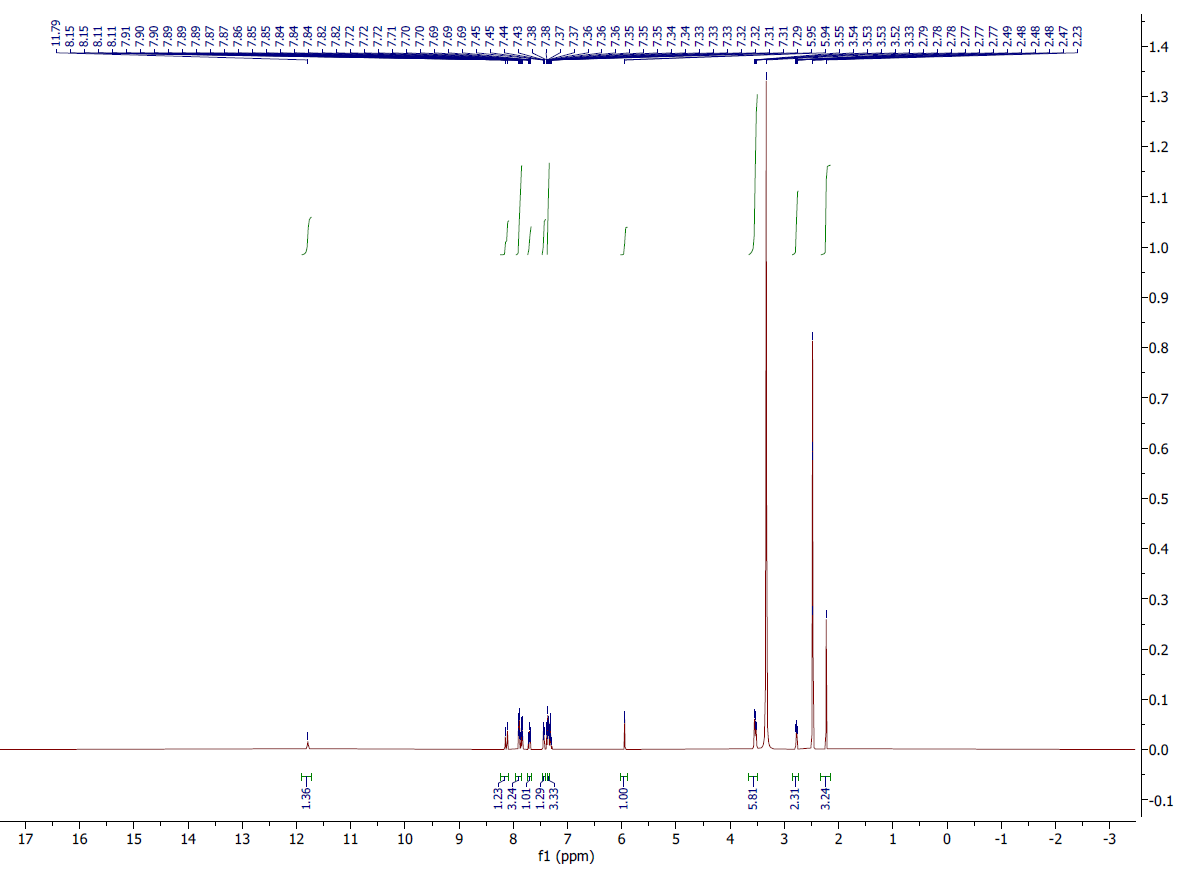

Figure S2 a: ^1^H NMR spectrum of compound **3b**


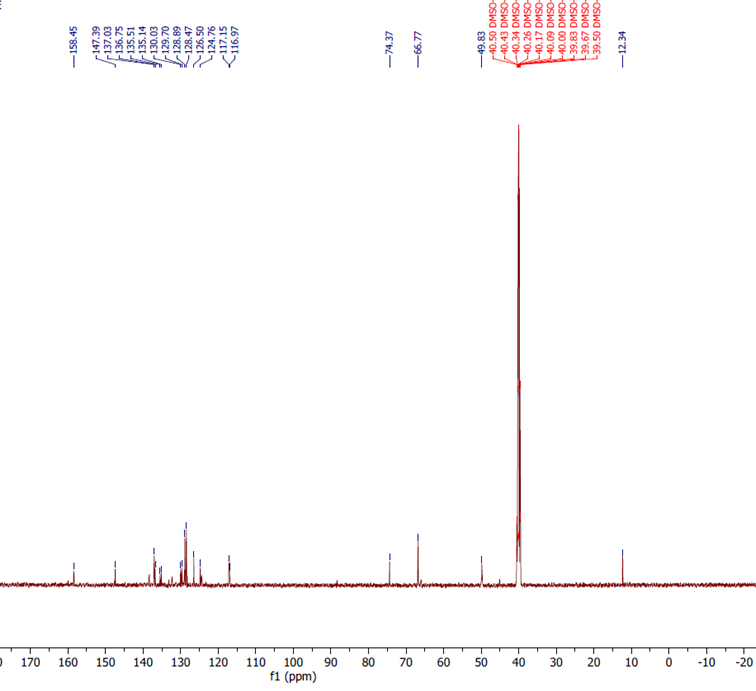

Figure S2 b: ^13^C NMR spectrum of compound **3b**


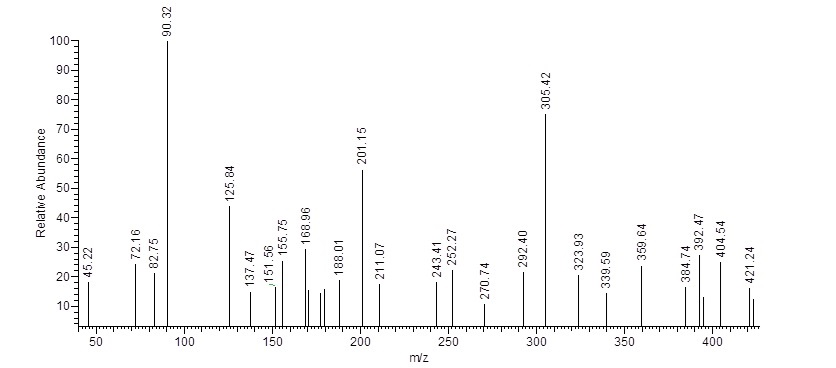


# :

Figure S2 c: Mass spectrum of compound **3b**

**Compound 3c**

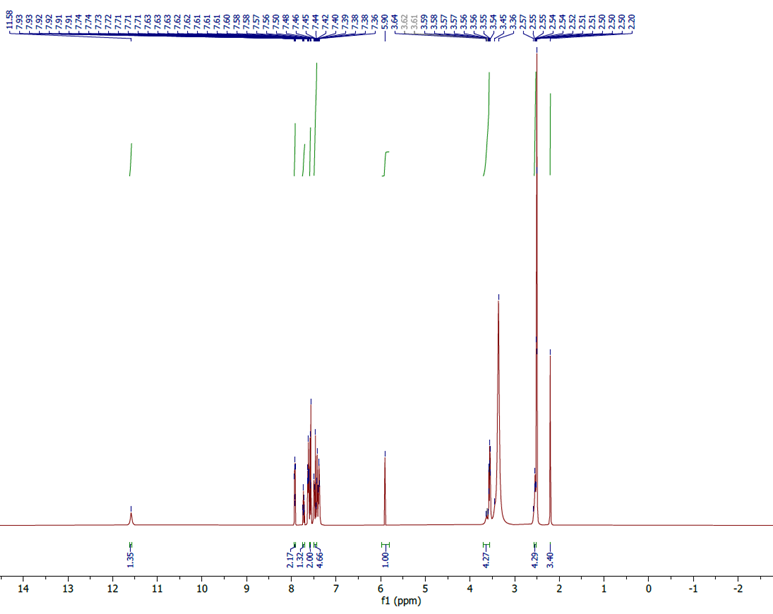

Figure S3 a: ^1^H NMR spectrum of compound **3c**

**
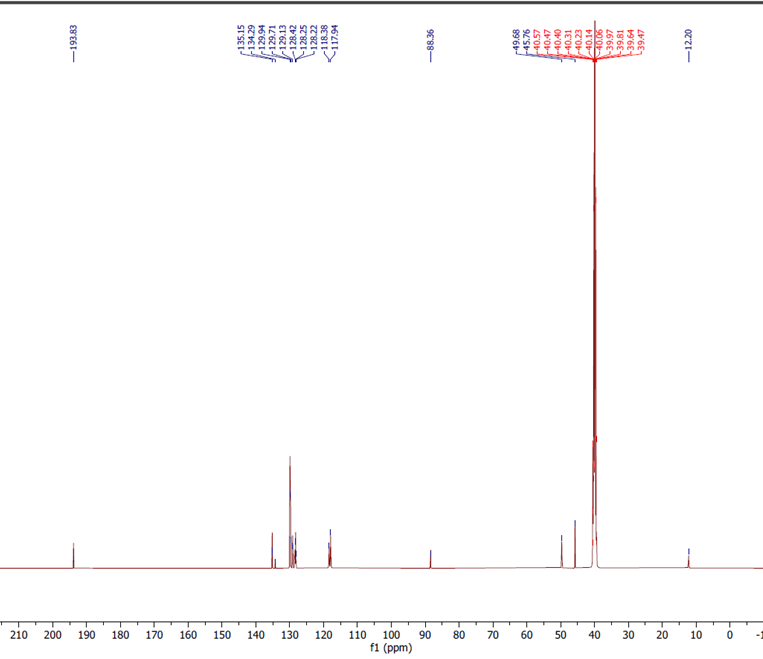
**

Figure S3 b: ^13^C NMR spectrum of compound **3c**


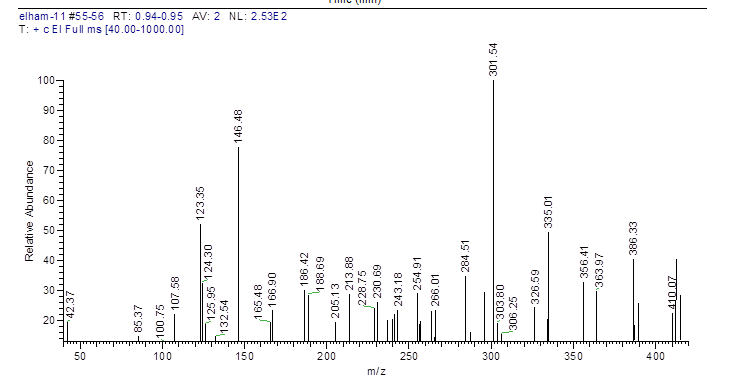


Figure S3 c: Mass spectrum of compound **3c**

**Compound 4a**

**
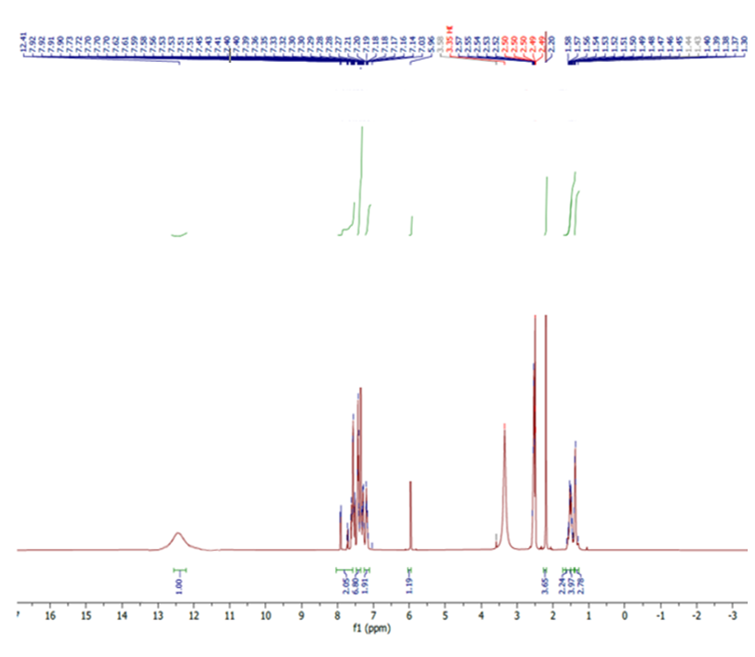
**

Figure S4 a: ^1^H NMR spectrum of compound **4a**

**
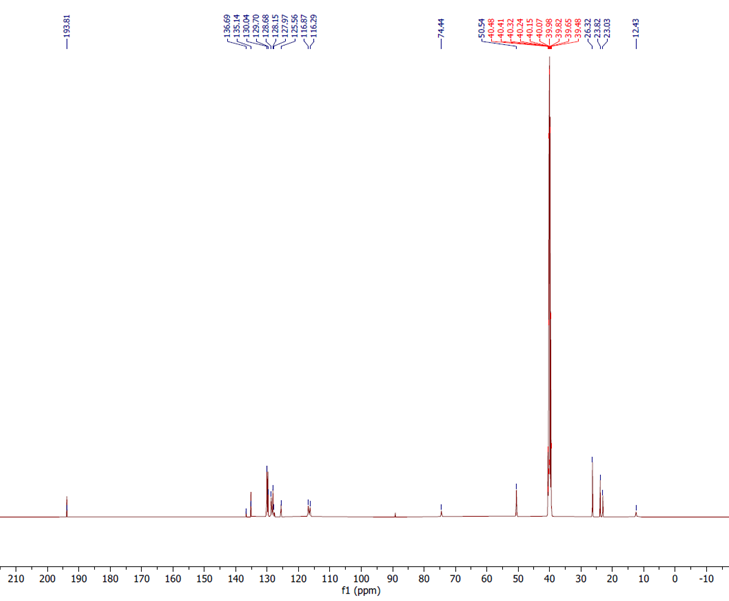
**

Figure S4 b: ^13^C NMR spectrum of compound **4a**


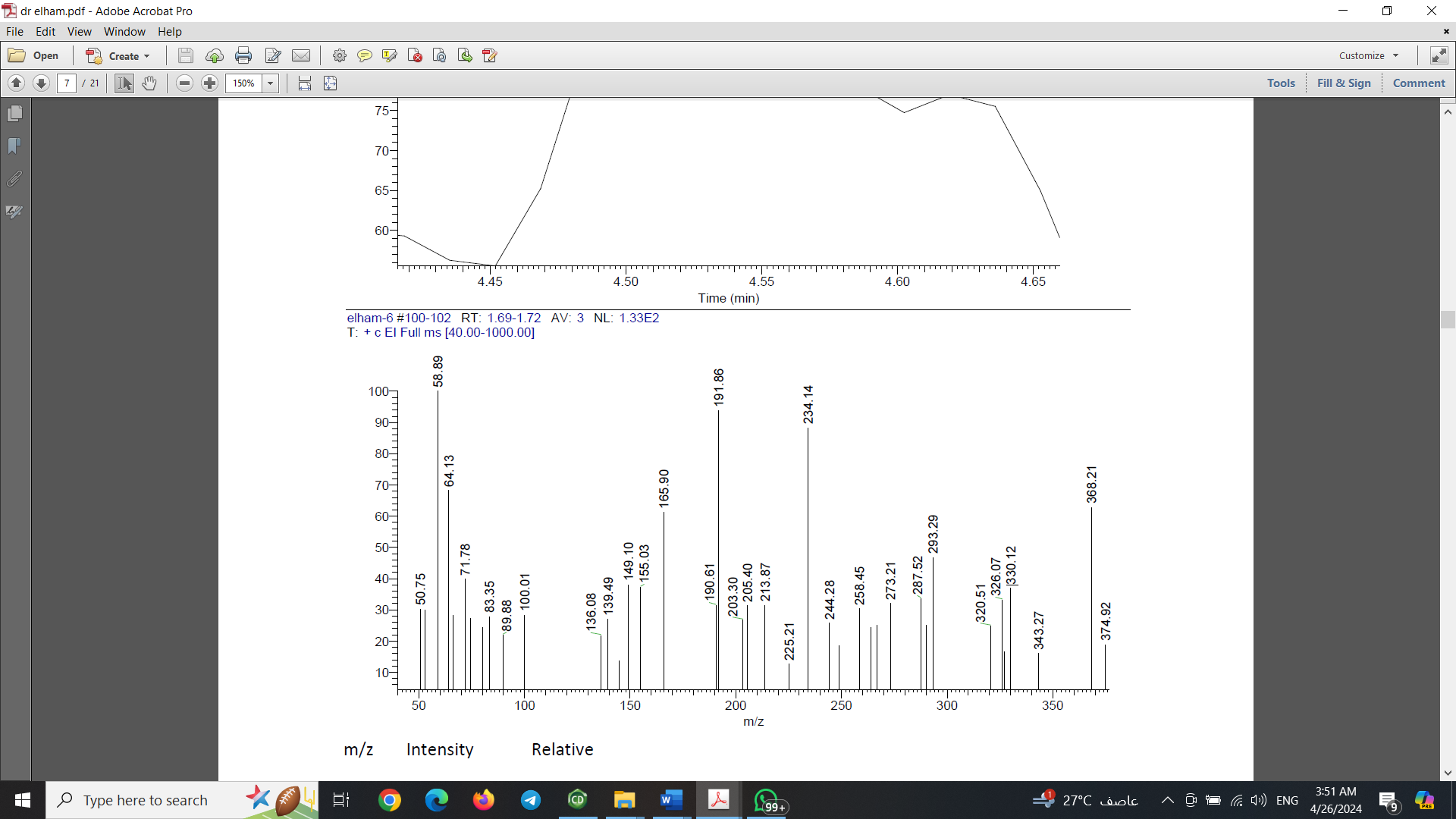


Figure S4 c: Mass spectrum of compound **4a**

**Compound 4b**

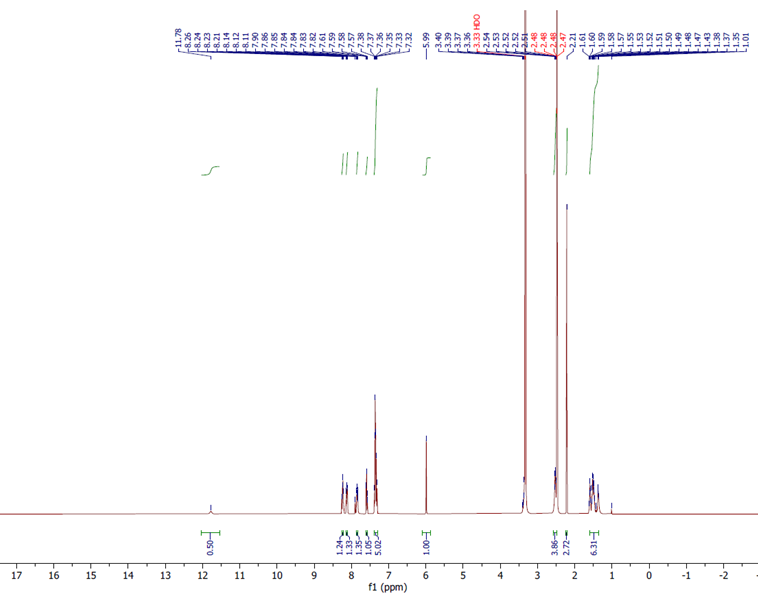

Figure S5 a: ^1^H NMR spectrum of compound **4b**


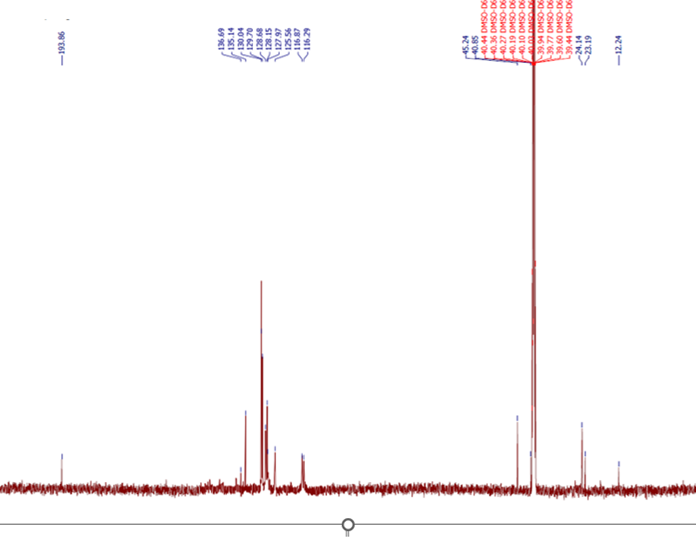

Figure S5 b: ^13^C NMR spectrum of compound **4b**


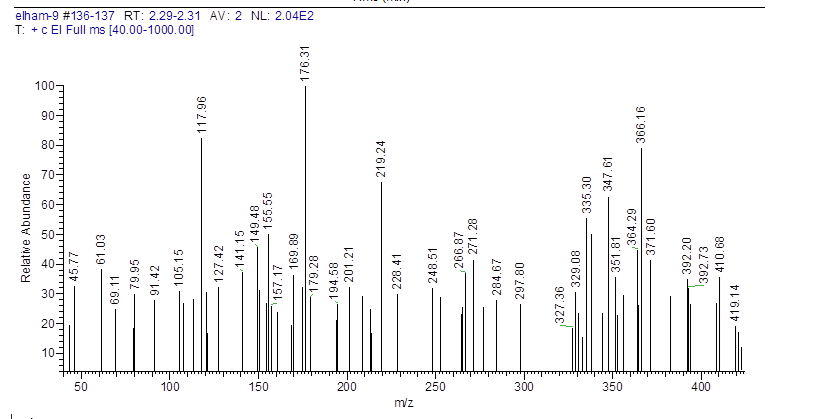


Figure S5 c: Mass spectrum of compound **4b**

**Compound 4c**

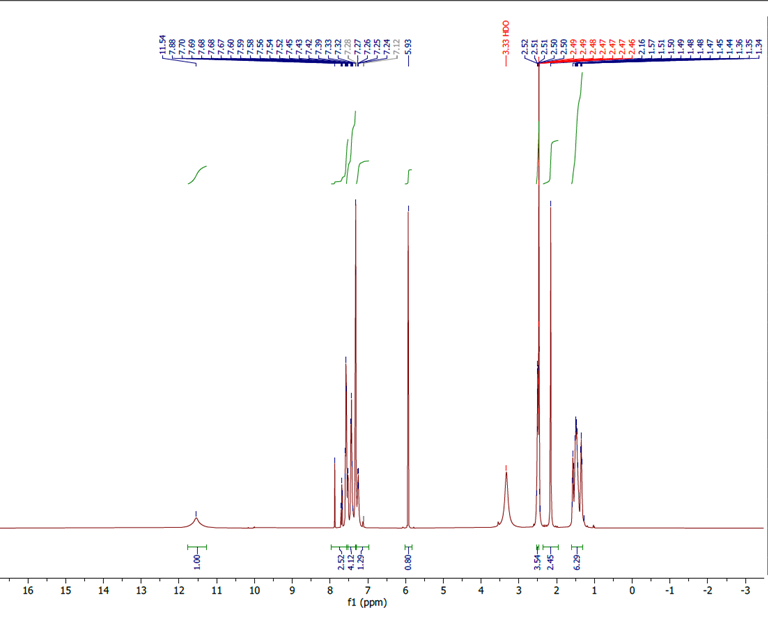

Figure S6 a: ^1^H NMR spectrum of compound **4c**


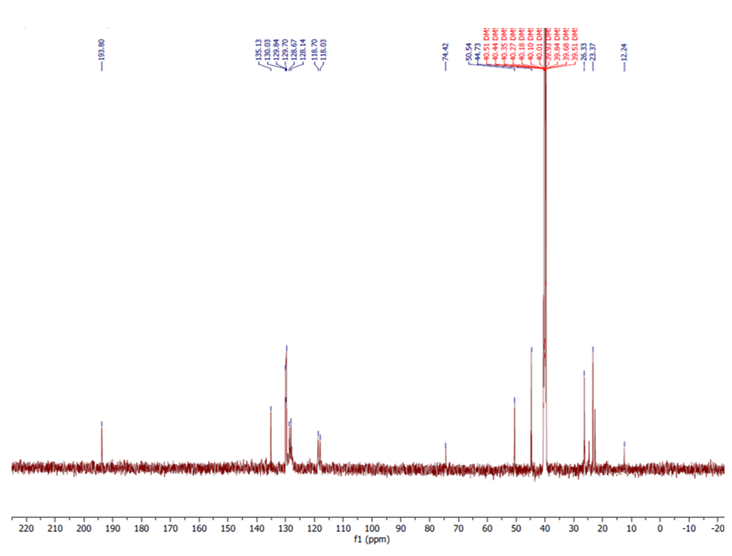

Figure S6 b: ^13^C NMR spectrum of compound **4c**

Figure S6 c: Mass spectrum of compound **4c**

**Compound 5a**

**
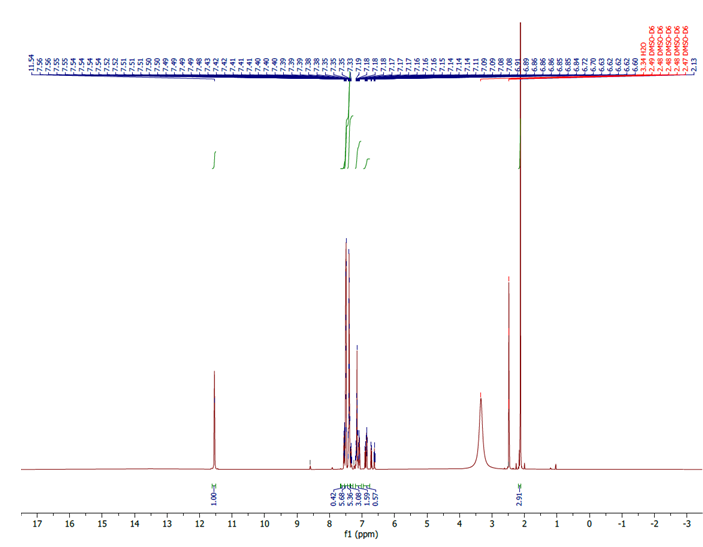
**

Figure S7 a: ^1^H NMR spectrum of compound **5a**

**
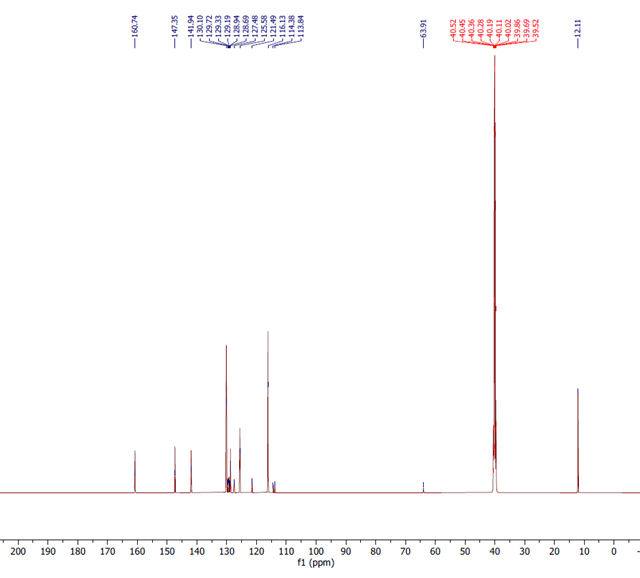
**

Figure S7 b: ^13^C NMR spectrum of compound **5a**


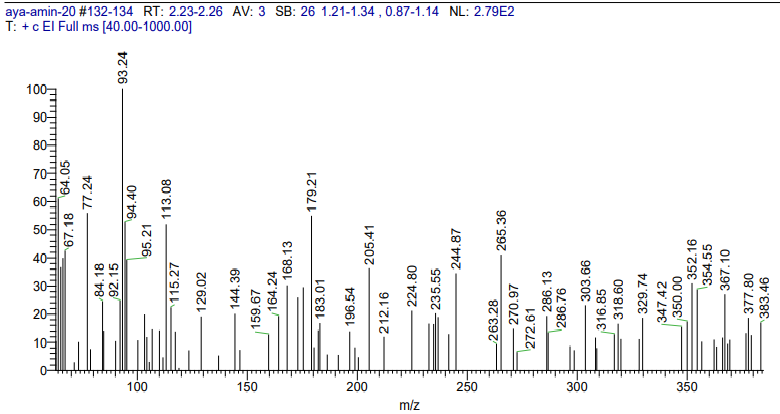


Figure S7 c: Mass spectrum of compound **5a**

**Compound 5b**

**
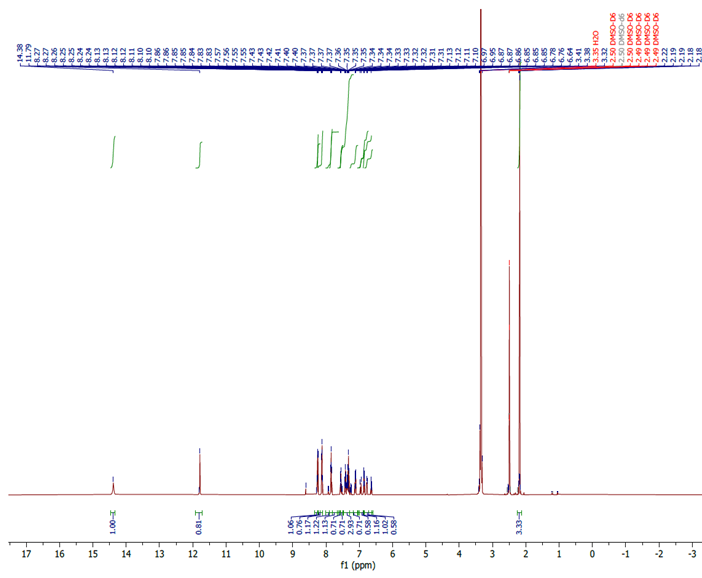
**

Figure S8 a: ^1^H NMR spectrum of compound **5b**

**
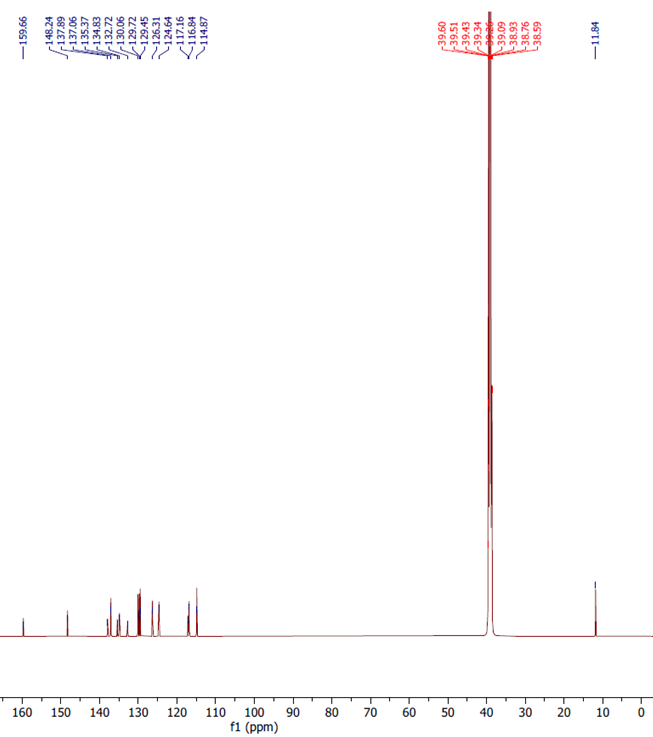
**

Figure S8 b: ^13^C NMR spectrum of compound **5b**


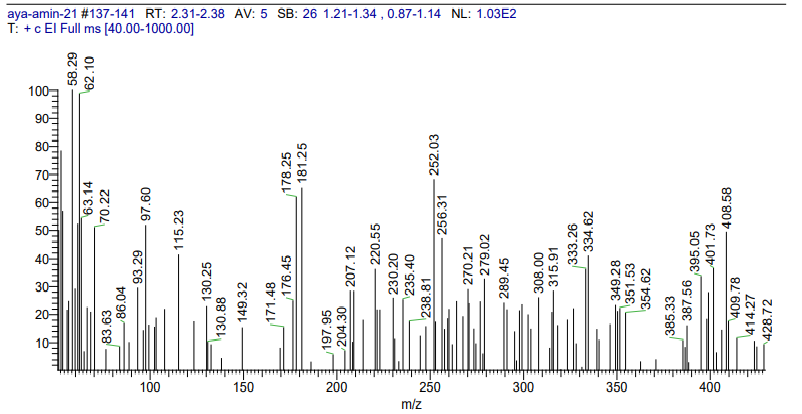


Figure S8 c: Mass spectrum of compound **5b**

**Compound 6a**


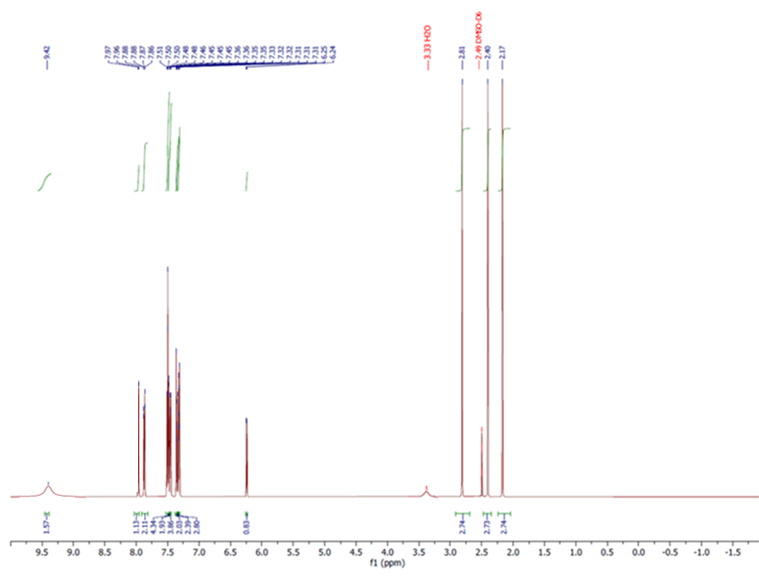

Figure S9 a: ^1^H NMR spectrum of compound **6a**


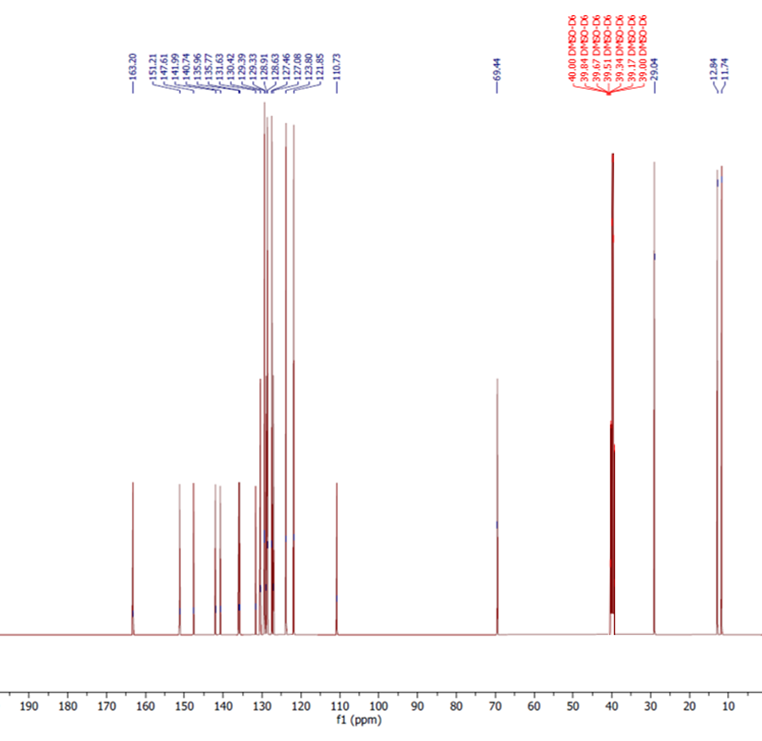

Figure S9 b: ^13^C NMR spectrum of compound **6a**


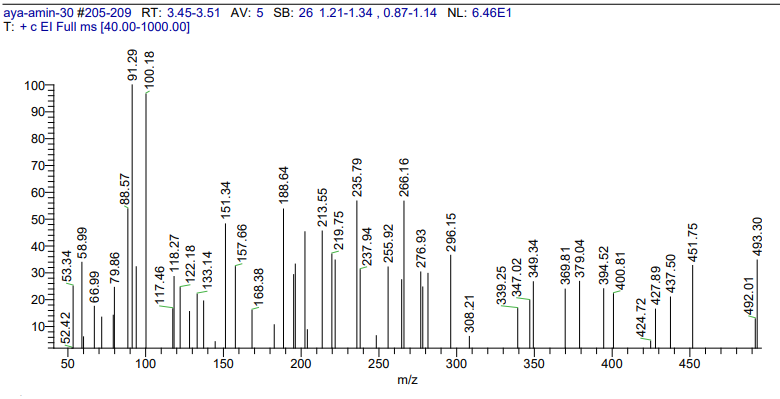


Figure S9 c: Mass spectrum of compound **6a**

 **Compound 6b**


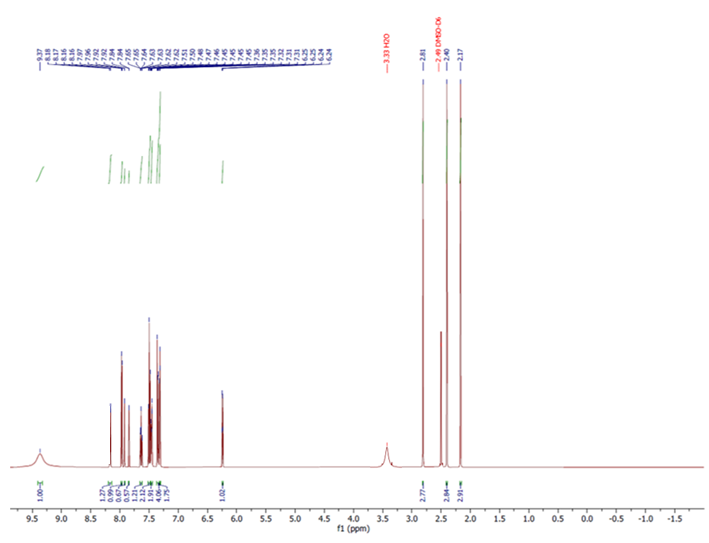

Figure S10 a: ^1^H NMR spectrum of compound **6b**


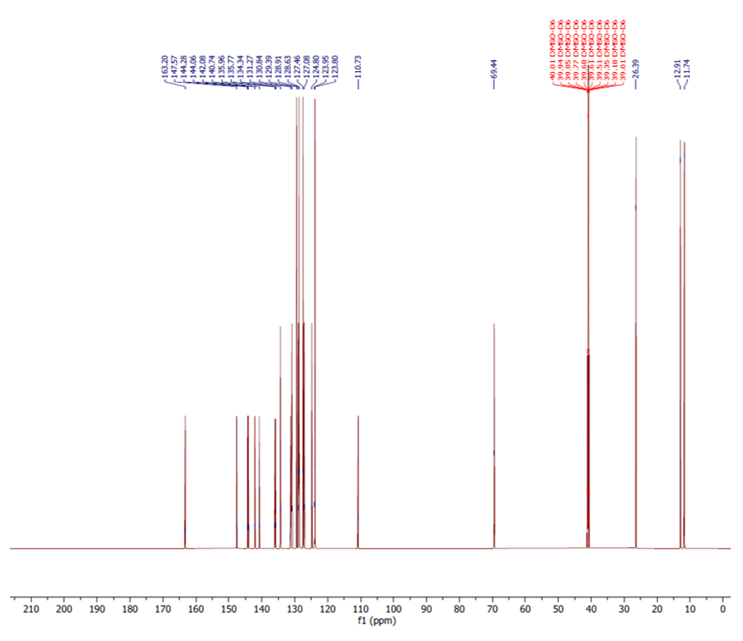

Figure S10 b: ^13^C NMR spectrum of compound **6b**


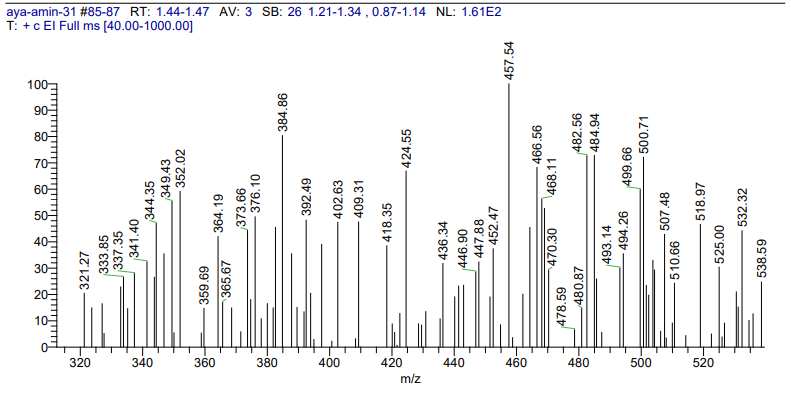


Figure S10 c: Mass spectrum of compound **6b**


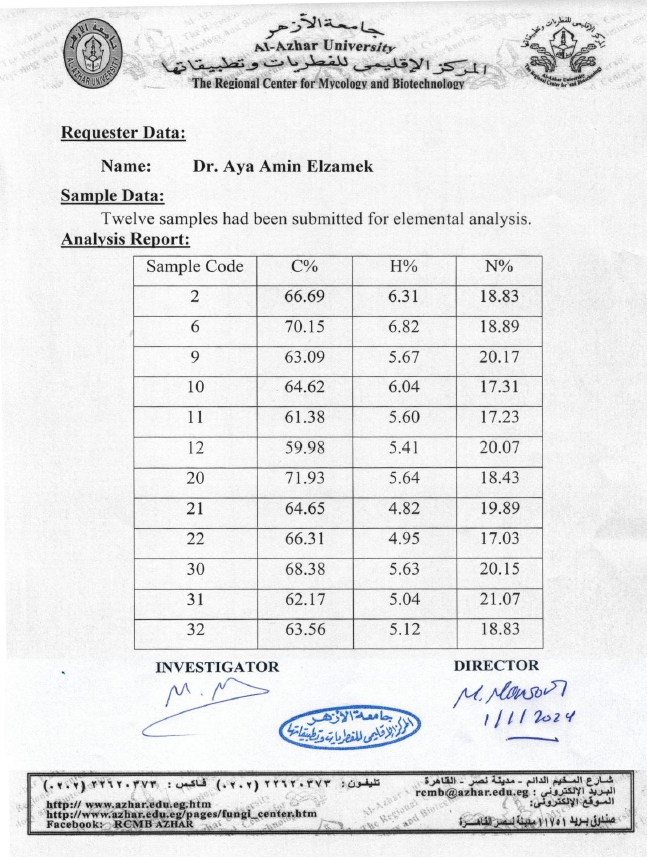


Figure S11: Elemental analysis of compounds

Table S1: Elemental analysis codes:

| **Compound** | **Code** |
| --- | --- |
| **3a** | 2 |
| **3b** | 12 |
| **3c** | 11 |
| **4a** | 6 |
| **4b** | 9 |
| **4c** | 10 |
| **5a** | 20 |
| **5b** | 21 |
| **6a** | 30 |
| **6b** | 31 |

**Supplementary part for biology work**


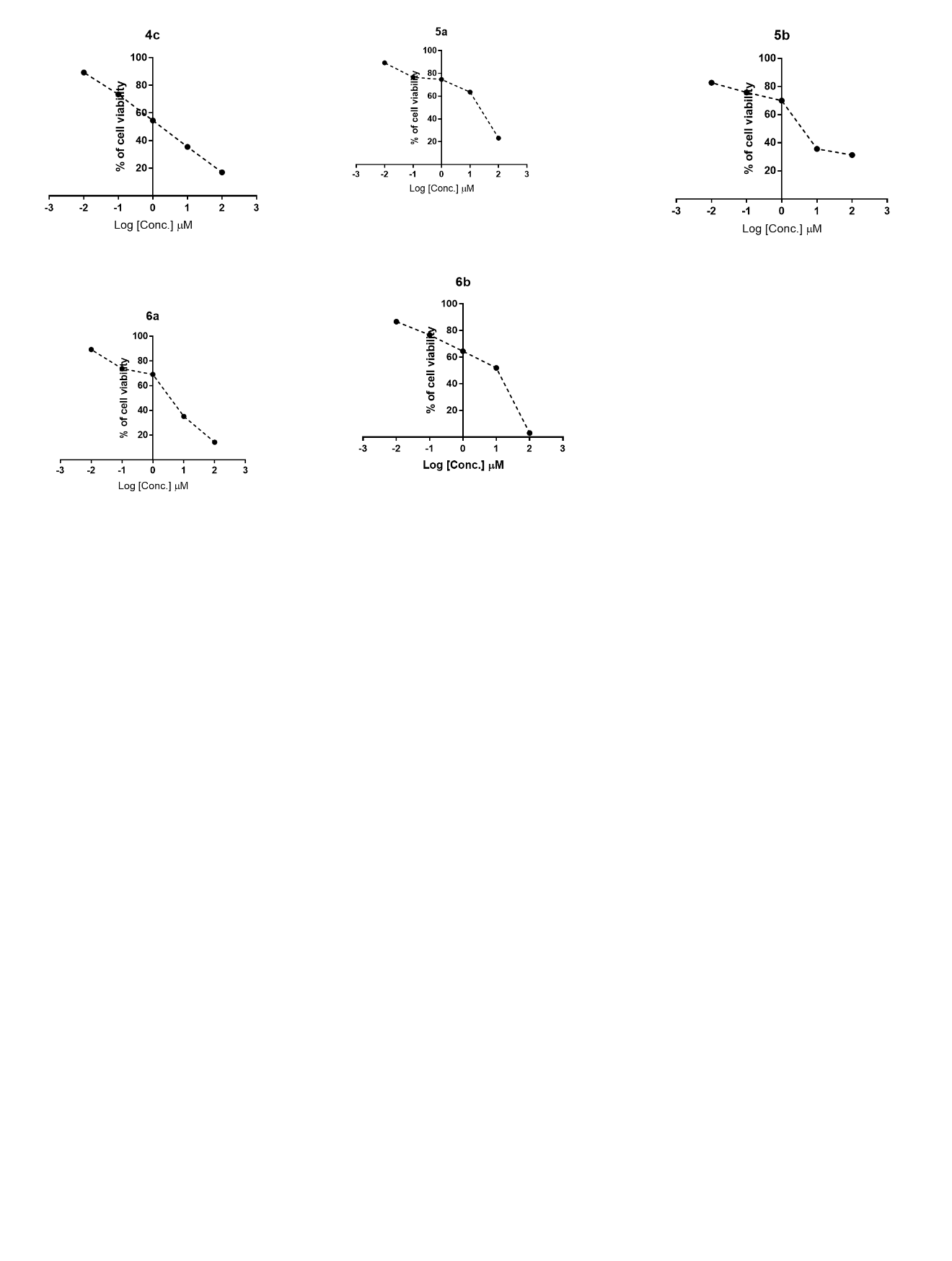

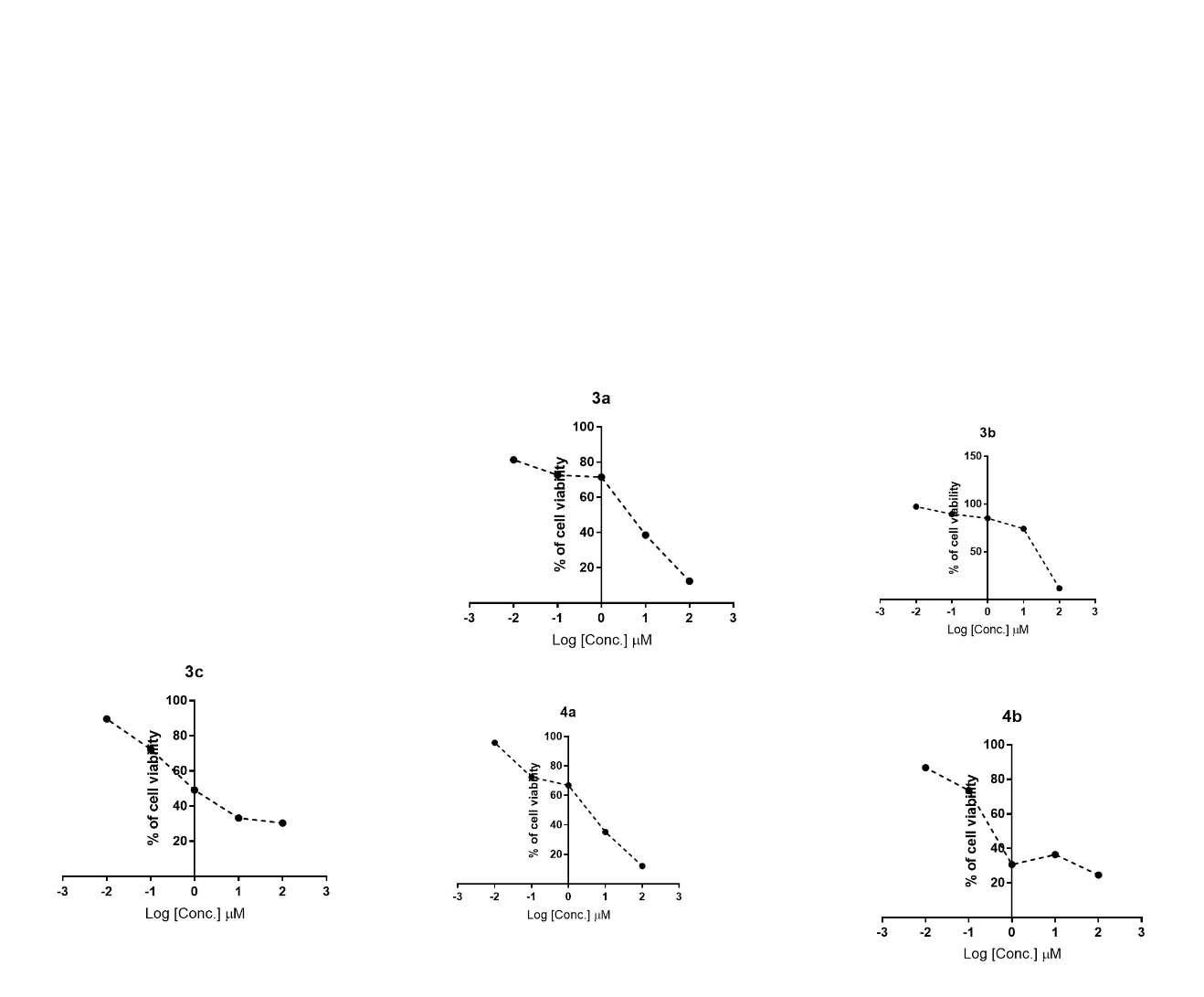


**Fig. S12.** Dose-response curve for the cytotoxicity of tested compounds (**10** compounds) against HepG2 cancer cells.


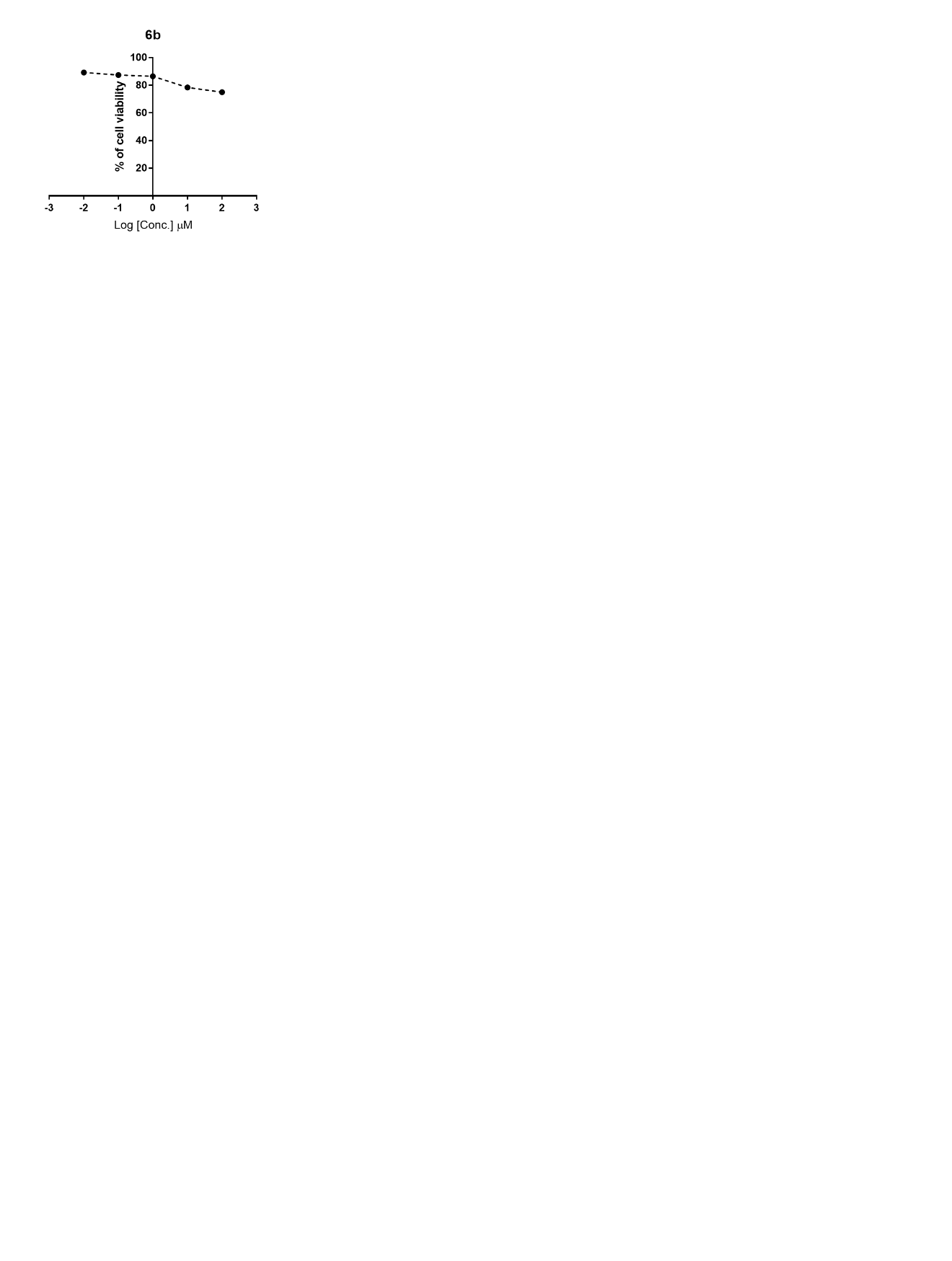

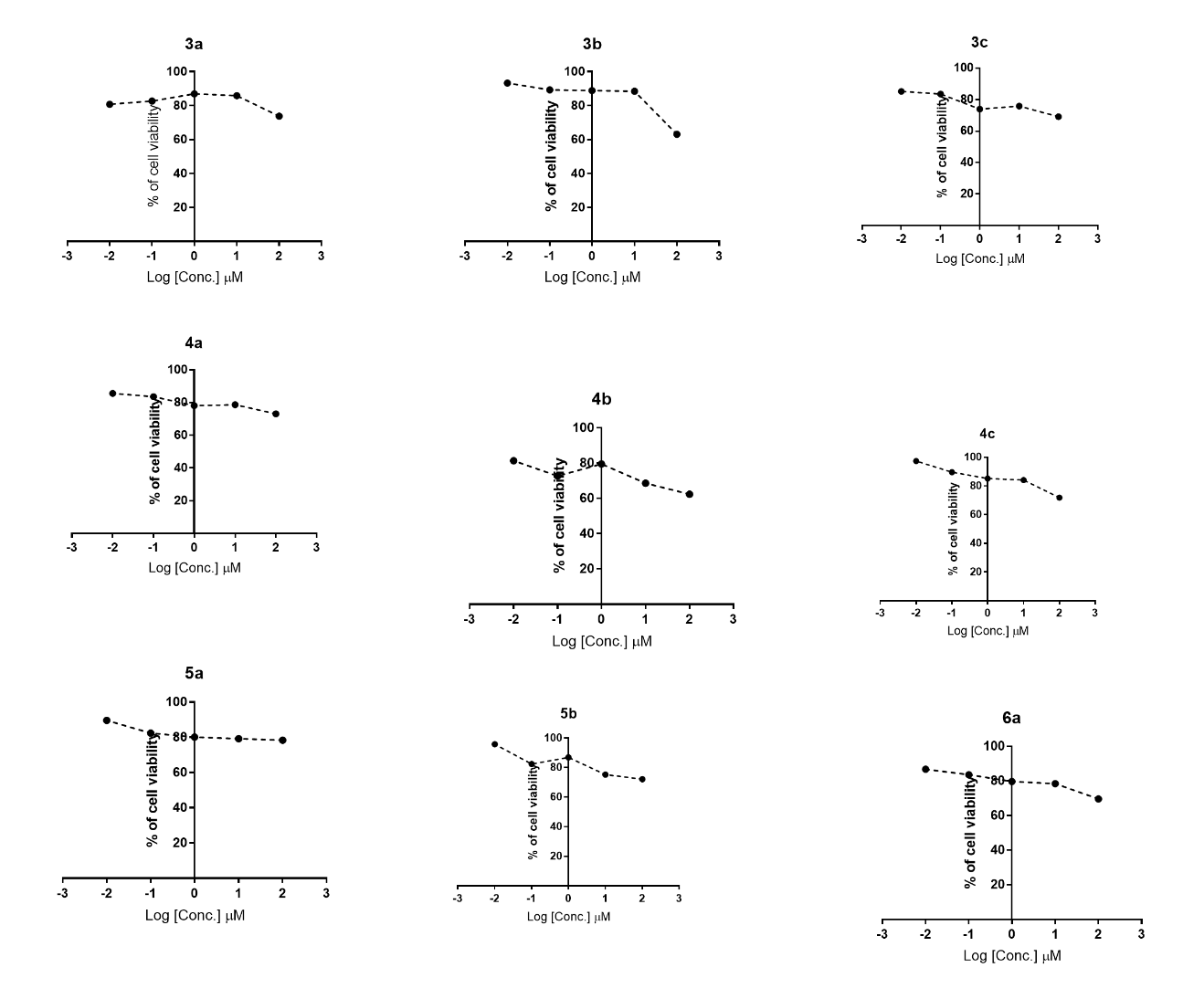


**Fig. S13.** Dose-response curve for the cytotoxicity of tested compounds (**10** compounds) against THLE2 normal liver cells.

**Researcher** : Dr.Aya Ayman email : [Aya.ayman1388@gmail.com](mailto:Aya.ayman1388@gmail.com) mob. 01202554451

Assay : Cell Cycle Analysis

Samples : 02 samples

cell line : ---

Ref. : ---

Date : 05-05-2023

Reader : BD FACSCalibur

Kit used : ab139418_Propidium Iodide Flow Cytometry Kit/BD

Solvent : DMSO

Assay samples : Cell culture

**Lab Report**

| **ser** | **Sample** | | **DNA content** | | | |  |
| --- | --- | --- | --- | --- | --- | --- | --- |
|  | **code** | **IC50**  **uM** | **%G0-G1** | **%S** | **%G2/M** | **Comment** |  |
| 1 | **s31/HepG2** | --- | 46.18 | 41.33 | 12.49 | cell growth arrest@ G1/S |  |
| 2 | **s20/HepG2** | --- | 49.18 | 35.21 | 15.61 | cell growth arrest@ G1 |  |
| 3 | **Cont.HepG2** | --- | 41.81 | 37.59 | 20.6 | --- |  |

| **s** | **code** | **conc** | **Apoptosis** | | | **Necrosis** |
| --- | --- | --- | --- | --- | --- | --- |
|  |  |  | Total | Early | Late |  |
| 1 | **s31/HepG2** | --- | **51.19** | **29.17** | **14.48** | **7.54** |
| 2 | **s20/HepG2** | --- | **42.95** | **26.77** | **11.55** | **4.63** |
| 3 | **Cont.HepG2** | --- | **2.47** | **0.72** | **0.23** | **1.52** |

**Detailed results**


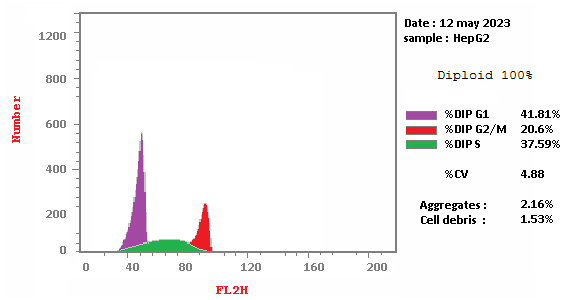


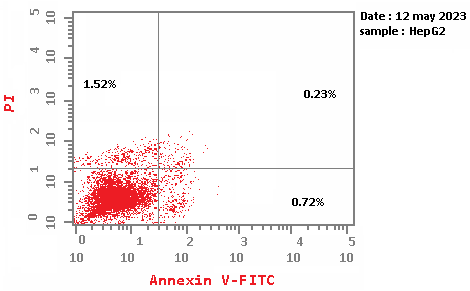


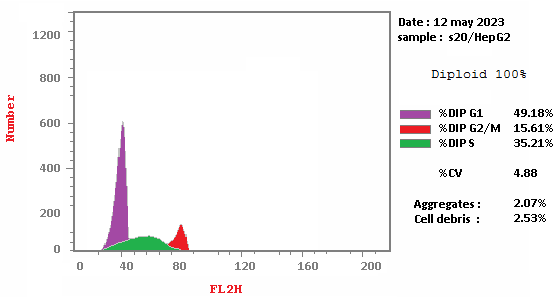


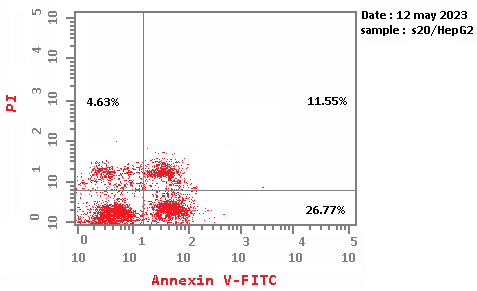


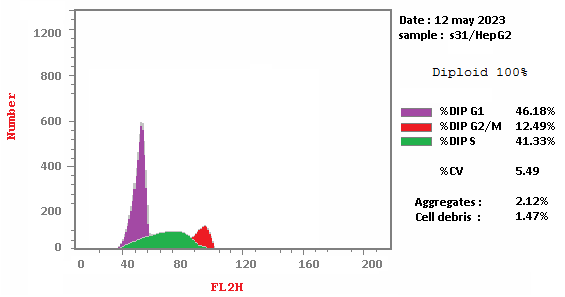


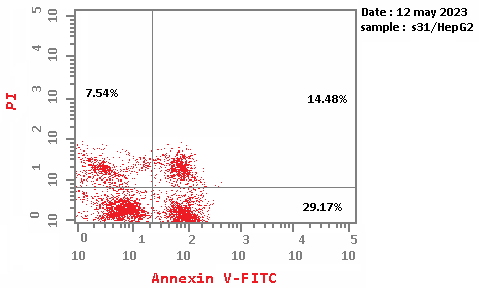


**Fig. S14.** Raw data of flow cytometry analysis; Annexin V/PI staining and Cell cycle analysis of compounds **5a** (s31) and **6b** (s20).
